# Host-Virus Protein Interaction Network Reveals the Involvement of Multiple Host Processes in the Life Cycle of Hepatitis E Virus

**DOI:** 10.1128/mSystems.00135-17

**Published:** 2018-01-23

**Authors:** Chandru Subramani, Vidya P. Nair, Saumya Anang, Sukhen Das Mandal, Madhu Pareek, Nidhi Kaushik, Akriti Srivastava, Sudipto Saha, Shalimar, Baibaswata Nayak, C. T. Ranjith-Kumar, Milan Surjit

**Affiliations:** a Virology Laboratory, Vaccine and Infectious Disease Research Centre, Translational Health Science and Technology Institute, NCR Biotech Science Cluster, 3rd Milestone, Faridabad-Gurgaon Expressway, Faridabad, Haryana, India; b Bioinformatics Centre, Bose Institute, Kolkata, West Bengal, India; c Department of Gastroenterology, All India Institute of Medical Sciences, Gautam Nagar, Ansari Nagar East, New Delhi, Delhi, India; Harvard Medical School

**Keywords:** hepatitis E virus, host-pathogen interactions, protein-protein interactions

## Abstract

Comprehensive knowledge of host-pathogen interactions is central to understand the life cycle of a pathogen and devise specific therapeutic strategies. Protein-protein interactions (PPIs) are key mediators of host-pathogen interactions. Hepatitis E virus (HEV) is a major cause of viral hepatitis in humans. Recent reports also demonstrate its extrahepatic manifestations in the brain. Toward understanding the molecular details of HEV life cycle, we screened human liver and fetal brain cDNA libraries to identify the host interaction partners of proteins encoded by genotype 1 HEV and constructed the virus-host PPI network. Analysis of the network indicated a role of HEV proteins in modulating multiple host biological processes such as stress and immune responses, the ubiquitin-proteasome system, energy and iron metabolism, and protein translation. Further investigations revealed the presence of multiple host translation regulatory factors in the viral translation/replication complex. Depletion of host translation factors such as eIF4A2, eIF3A, and RACK1 significantly reduced the viral replication, whereas eIF2AK4 depletion had no effect. These findings highlight the ingenuity of the pathogen in manipulating the host machinery to its own benefit, a clear understanding of which is essential for the identification of strategic targets and development of specific antivirals against HEV.

**IMPORTANCE** Hepatitis E virus (HEV) is a pathogen that is transmitted by the fecal-oral route. Owing to the lack of an efficient laboratory model, the life cycle of the virus is poorly understood. During the course of infection, interactions between the viral and host proteins play essential roles, a clear understanding of which is essential to decode the life cycle of the virus. In this study, we identified the direct host interaction partners of all HEV proteins and generated a PPI network. Our functional analysis of the HEV-human PPI network reveals a role of HEV proteins in modulating multiple host biological processes such as stress and immune responses, the ubiquitin-proteasome system, energy and iron metabolism, and protein translation. Further investigations revealed an essential role of several host factors in HEV replication. Collectively, the results from our study provide a vast resource of PPI data from HEV and its human host and identify the molecular components of the viral translation/replication machinery.

## Abstract (precis)

Hepatitis E virus (HEV) is a pathogen that is transmitted by the fecal-oral route. Owing to the lack of an efficient laboratory model, the life cycle of the virus is poorly understood. During the course of infection, interactions between the viral and host proteins play essential roles, a clear understanding of which is essential to decode the life cycle of the virus. In this study, we identified the direct host interaction partners of all HEV proteins and generated a PPI network. Our functional analysis of the HEV-human PPI network reveals a role of HEV proteins in modulating multiple host biological processes such as stress and immune responses, the ubiquitin-proteasome system, energy and iron metabolism, and protein translation. Further investigations revealed an essential role of several host factors in HEV replication. Collectively, the results from our study provide a vast resource of PPI data from HEV and its human host and identify the molecular components of the viral translation/replication machinery.

## INTRODUCTION

In the evolutionary race for survival, a successful pathogen encodes suitable strategies that not only enable it to survive under adverse host conditions but also allow it to usurp the host machineries for its own survival. A crucial event in the life of an RNA virus pertains to its ability to utilize the host resources to synthesize its own proteins and replicate its genome (1). Many viruses such as poliovirus, cricket paralysis virus, hepatitis A virus, and hepatitis C virus (HCV) contain internal ribosome entry sites (IRES), which utilize host translation factors to selectively initiate viral protein synthesis (2–5). Influenza virus polymerase binds eIF4G to promote viral protein translation (6). HCV NS5A, herpes simplex virus 1 (HSV-1) Us3, and human cytomegalovirus (HCMV) UL38 phosphorylate 4E-BP1, indirectly promoting viral translation (7–9).

Hepatitis E virus (HEV) is a positive-strand RNA virus that infects humans and many other animals, usually causing an acute self-limiting hepatitis (10). In organ-transplant patients or immunocompromised individuals, it causes chronic infection (11). In HEV-infected pregnant women, the mortality rate is up to ~30% (12). All HEV genotypes contain three open reading frames (ORFs), namely, ORF1, ORF2, and ORF3. Recently, we identified an additional ORF, ORF4, in genotype 1 HEV (g-1 HEV), which is located in the +1 reading frame position (with respect to ORF1) within ORF1 and produces a 158-amino-acid (aa)-long protein by an internal translation mechanism. ORF1 encodes a polyprotein containing distinct domains such as methyltransferase (Met), Y domain (Y), papain-like cysteine protease (PCP), V domain (V), X domain (X), helicase (Hel), and RNA-dependent RNA polymerase (RdRp). The ORF2 gene codes for the ORF2 protein, which is the major capsid protein of the virus, and the ORF3 gene codes for the ORF3 protein, which plays an important role in virus egress and is also believed to be involved in multiple signal transduction processes (13).

No conclusive data exist regarding processing of ORF1 polypeptide into individual domains. Shorter fragments corresponding to processed ORF1 products were detected in some previous studies (14, 15), whereas no processing was observed in a few other reports (16, 17). However, a recent study reported that HEV protease (PCP) possesses chymotrypsin-like enzyme activity and is able to process parts of ORF1 polypeptide, as well as ORF2 protein, *in vitro*, indicating that it might perform similar functions in an infection scenario (18). It is noteworthy that all the studies pertaining to ORF1 processing have been carried out in heterologous systems owing to lack of an efficient laboratory model for HEV. Hence, the ORF1 processing that occurs during the course of infection remains to be investigated. Functional characteristics of a few ORF1 domains have been analyzed to some extent in cultured mammalian cells, with results which demonstrate that viral PCP deubiquitinates interferon-stimulated gene-15, retinoic-acid-inducible gene-I, and TANK binding kinase-1; X domain inhibits interferon-regulatory factor-3 phosphorylation and possesses de-ADP-ribosylation and de-MARylation activity (19–21). The ORF3 protein interacts with multiple host proteins, including TSG101, microtubules, alpha-1-microglobulin, bikunin, fibrinogen, hemopexin, and CIN85 (22). The interaction of ORF3 and TSG101 was shown to be essential for g-3 HEV release (23). Proteomic analyses of plasma from g-1 HEV-infected humans, g-3 HEV-infected swine liver cells, and g-4 HEV-infected A549 cells have identified the differential protein expression profiles in those samples (24–26).

Limited information exists regarding the mechanism of hepatitis E viral replication. The host ubiquitin-proteasome system has been reported to play an essential role in HEV replication, and knockdown of the host eIF4F complex was shown to inhibit HEV replication, although the underlying mechanisms remain unknown (27, 28). We reported earlier the essential role of host eEF1α1 and viral ORF4 protein in mediating the optimal activity of viral RdRp (13). Additional viral and host factors are likely to be essential components of the viral replication complex. In the absence of an efficient and handy model to study the virus in the laboratory, it is impossible to employ genome-wide knockdown strategies to identify the host components involved in the viral life cycle.

In order to understand the life cycle of HEV, we identified the host interaction partners of all the viral proteins and generated a protein-protein interaction (PPI) network for HEV and its human host. Bioinformatics analyses of the primary and secondary interaction partners revealed the predominance of factors involved in metabolic, proteasome, and protein translation pathways. The significance of these interactions in the context of the life cycle of HEV was explored.

## RESULTS

### Identification of direct host interaction partners of the proteins encoded by the hepatitis E virus.

**FIG 1:**
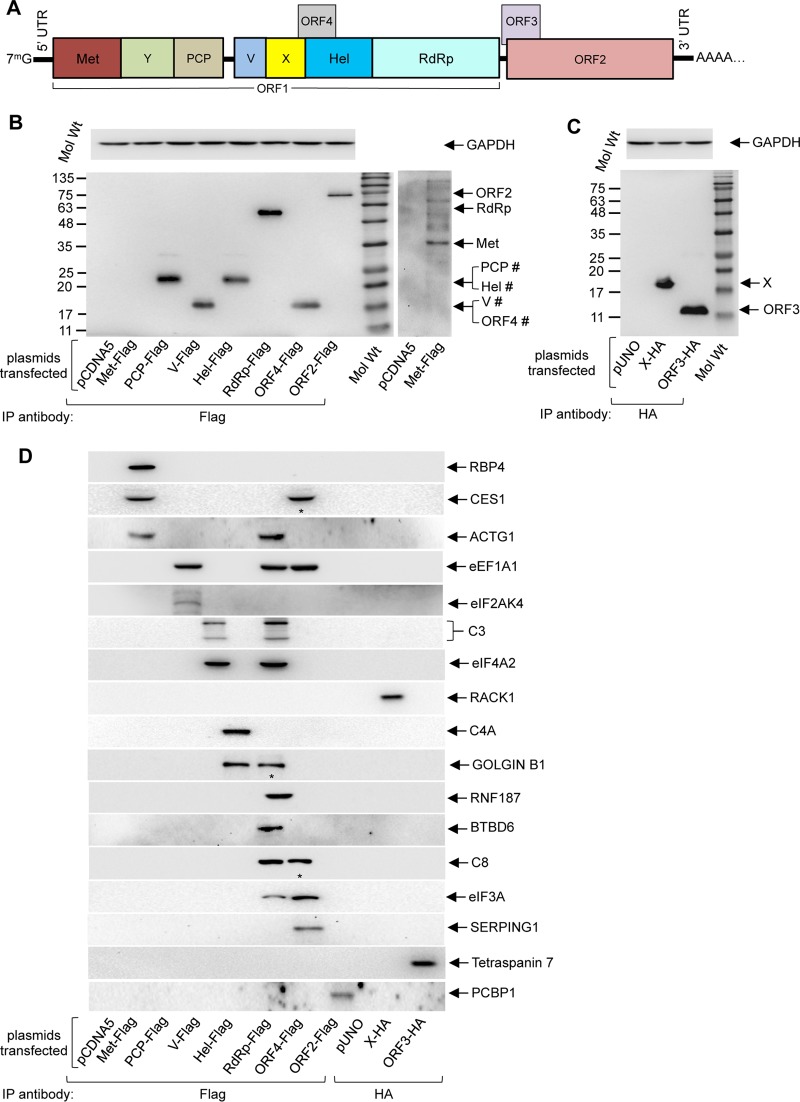
Identification of the host interaction partners of HEV proteins in the human liver and fetal brain cDNA libraries. (A) Schematic of the g-1 HEV genome and of the proteins encoded by it. UTR, untranscribed region. (B) (Upper panel) Western blot showing GAPDH protein level in Huh7 cell extract expressing the indicated Flag-tagged viral proteins. (Lower left panel) Coimmunoprecipitation of Flag-tagged viral proteins expressed in Huh7 cells using Flag-agarose beads and Western blotting using anti-Flag antibody. The right panel shows a higher exposure of the same blot (cropped) to reveal the signal of Met-Flag protein. The crosshatch symbol (#) denotes that PCP and Hel and V and ORF4 migrate at similar sizes. Mol Wt, molecular weight. (C) (Upper panel) Western blot showing GAPDH protein level in Huh7 cell extract expressing the indicated HA-tagged viral proteins. (Lower panel) Coimmunoprecipitation of HA-tagged viral proteins expressed in Huh7 cells using HA-agarose beads and Western blotting using anti-HA antibody. (D) Aliquots of CoIP samples (shown in panels B and C) were immunoblotted using the indicated antibodies.

A GAL4-based yeast two-hybrid (Y2H) system was used to screen the direct host interaction partners of seven distinct ORF1 domains and three mature proteins (ORF2, ORF3, and ORF4) of g-1 HEV. Viral proteins (Fig. 1A) were cloned into the bait vector (pGBKT7), and their self-activation potential was tested in the Y2H gold strain (data not shown). Pretransformed human liver and fetal brain cDNA libraries were mated with the baits expressed in the Y2H gold strain, with average mating efficiencies of 5.1% and 3.4%, respectively (see Table S1 in the supplemental material). The average numbers of clones screened by each bait against the fetal brain and liver libraries were 1.4 million and 2.5 million, respectively (Table S3). Diploids were tested for reporter gene activation, followed by isolation and verification of prey plasmids by restriction mapping (Table 1). Prey clones with unique restriction patterns were retransformed into the Y2H gold strain along with the empty bait vector or the corresponding viral-protein-expressing bait vector to reconfirm the interactions (Table S2A). Prey clones lacking self-activation and activating all four reporters in the presence of the bait were sequenced. A prey was considered a bonafide interaction partner only if the insertion contained an in-frame fusion of the protein coding sequence with the binding domain (BD) (Table 1; see also Table S1). Note that only the fetal brain library was screened by us against the ORF3 protein; the results identified 11 unique host proteins. Since many reports exist for human liver library screening against ORF3, these data were sourced from the literature (32 host proteins) (22). A human liver cDNA library was screened against ORF4 in this study, and data for fetal brain library screening against RdRp was sourced from our previous report (13). Collectively, 138 unique host proteins (H_HEV_) involved in 169 PPIs were identified (Table 2). A total of 84% of the interaction partners isolated from the fetal brain library were expressed in the liver as well, as evident from the comparison of transcript levels of those data sets in human liver and fetal brain using the Human Protein Atlas database (http://www.proteinatlas.org/) (Table S4). A total of 24 host proteins are targeted by two or more viral proteins (Table 3), suggesting their importance in the formation of virus-host multiprotein complexes.

10.1128/mSystems.00135-17.6 TABLE S1  Estimation of efficiency of mating of bait and prey strains. Download TABLE S1, PDF file, 0.1 MB. Copyright © 2018 Subramani et al. 2018 Subramani et al. This content is distributed under the terms of the Creative Commons Attribution 4.0 International license.

10.1128/mSystems.00135-17.7 TABLE S2  Y2H assay to confirm the interaction partners (H_HEV_) of HEV proteins. Download TABLE S2, XLSX file, 0.04 MB. Copyright © 2018 Subramani et al. 2018 Subramani et al. This content is distributed under the terms of the Creative Commons Attribution 4.0 International license.

10.1128/mSystems.00135-17.8 TABLE S3  Sequencing data analysis of the host interaction partners of g-1 HEV proteins. Download TABLE S3, XLSX file, 0.02 MB. Copyright © 2018 Subramani et al. 2018 Subramani et al. This content is distributed under the terms of the Creative Commons Attribution 4.0 International license.

10.1128/mSystems.00135-17.9 TABLE S4  Comparison of levels of expression of H_HEV_ proteins identified by fetal brain library screening in human brain and liver tissues. Download TABLE S4, XLSX file, 0.01 MB. Copyright © 2018 Subramani et al. 2018 Subramani et al. This content is distributed under the terms of the Creative Commons Attribution 4.0 International license.

**TABLE 1:** Comprehensive summary of g-1 HEV and human cDNA library screening^a^

Viral bait	LTHA^−^	LTHA^−^ + A^r+^ + X α-Gal	LT^−^ + X α-Gal (3 times)	No. of plasmids	Unique restriction pattern	No. of colonies after retransformation	Unique coding sequence in frame with BD
Fetal brain cDNA library screening							
Met	43	35	30	29	16	10	4
Y	1	0	0	0	0	0	0
PCP	89	50	27	25	21	12	2
V	237	112	71	31	7	5	3
X	96	57	54	52	11	8	5
Hel	13	1	1	0	0	0	0
RdRp	300^b^	300^c^	255	225	174	140	21
ORF2	5	2	2	1	1	1	1
ORF3	330	180	110	91	65	44	11
ORF4	144	135	108	105	62	50	8
Liver cDNA library screening							
Met	105	50	32	27	23	22	15
Y	38	7	7	7	4	4	4
PCP	180	95	80	76	27	11	3
V	233	150	140	117	24	12	6
X	74	57	57	52	20	17	6
Hel	117	23	22	22	20	12	9
RdRp	244^b^	222^c^	203	199	112	60	20
ORF2	15	8	8	6	6	2	1
ORF4	302	209	164	159	89	55	18

a BD, binding domain; L, leucine; T, tryptophan; H, histidine; A, adenine; A^r+^, aureobasidin A; α-Gal, alpha-galactosidase; −, deficiency in the medium; +, supplemented in the medium.

b For RdRp, after mating cells were plated on LTHA^−^ + 10 mM 3-AT (3-amino 1,2,4 triazole).

c For RdRp, 10 mM 3-AT was included in the LTHA^−^ + A^r+^ + X α-Gal.

**TABLE 2:** List of primary interaction partners (H_HEV_) of HEV proteins^a^

Viral protein	No. of host interaction partners	Host proteins (gene symbol)
Primary interaction partners of g-1 HEV		
Met	19	APCS, ACTG1, ACY1, ALDH1L1, ALDOB, AOX1, CES1, CTSF, DBF4B, FNDC1, GPRC5C, HEMK1, KLHDC2, MAP2K5, PPIE, PSMB4, RBP4, SLC27A5, TUBB2A
Y	4	DNAJA3, MAP1S, TIMM50, TPST2
PCP	5	EEF1A1, EIF2AK4, PCDH8, RNF168, SLC22A12^b^
V	9	ALB, BIRC6, BTBD6, C3, EIF4A2, FUS, MT-CO1, MT-ND1, UBB^b^
X	11	AZGP1, RBP4, MT-ND4L, SLC35A2, GNB2L1, ALDOB, PSMB1, DAZAP2, C1orf61, DNAJB5, KREMEN1
Hel	9	A2M, ACY1, ALDH1L1, C4A, GNB2, GOLGB1, HEMK1, TF, TM4SF4^b^
RdRp	41	ACTG1, ADH1B, ALB, ALDOB, ALS2CL, APLP1, ATP9B, BTBD6, C3, C8A, CIRBP, COG1, DCXR EEF1A1, EIF3A,^b^ EIF4A2, FBOX31, FEZ1, GBE1,^b^ H2AFY2, HN1, HP, LSAMP, MACF1, MAP1B, MARCKSL1, MT-CO1, NEIL1, NR2F6, PCDL, PEBP1, PSAP, RNF187, RTN1, SARAF, SLC2A1, TF, TMX2, TUBB, VTN, ZDHHC6
ORF2	2	PCBP1, RPL29
ORF3	11	BRD2, C1orf61, DBF4B, ITM2C, MAD2L1, MCOLN1, NRXN2, SDK2, TSG101, TSPAN7, U2SURP
ORF4	26	ALDOB, APOH, ASGR2, AZGP1, AZIN1, CLPP, CP, CSAD, CTSD, EEF1A1, EIF3A, EPS8, FAM35A, FGA, FGB, GRB2, HNRNPLL, HP, HRG, KIAA1191, MPP1, MT-ATP6, MT-CO2, NPLOC4, SERPING1, SLC26A10, TEKT4, TUBB
		
Primary interaction partners of g-3 HEV		
Met	11	ACTG1, ACY1, ALDH1L1, AOX1, CES1, FNDC1, HEMK1, MAP2K5, PSMB4, RBP4, TUBB2A
Y	1	MAP1S
PCP	2	EEF1A1, SLC22A12
V	9	ALB, BIRC6, BTBD6, C3, EIF4A2, FUS, MT-CO1, MT-ND1, UBB
X	10	ALDOB, AZGP1, DAZAP2, DNAJB5, GNB2L1, KREMEN1, MT-ND4L, PSMB1, RBP4, SLC35A2
Hel	9	A2M, ACY1, ALDH1L1, C4A, GNB2, GOLGB1, HEMK1, TF, TM4SF4
RdRp	23	ACTG1, ALB, ALS2CL, BTBD6, C3, DCXR, EEF1A1, EIF3A, EIF4A2, FBXO31, FEZ1, GBE1, H2AFY2, HN1, MACF1, MAP1B, PSAP, RNF187, SARAF, SLC2A1, TUBB, VTN, ZDHHC6
ORF2	2	PCBP1, RPL29
ORF3	11	BRD2, C1orf61, DBF4B, ITM2C, MAD2L1, MCOLN1, NRXN2, SDK2, TSG101, TSPAN7, U2SURP
		
Primary interaction partners of g-1 HEV ORF1 polypeptide		
ORF1		AY1, A2M, ACTG1, ACY1, ADH1B, ALDH1L1, ALDOB, ALS2CL, AOX1, APCS, APLP1, ATP9B, AZGP1, BIRC6, BTBD6, C1orf61, C3, C4A, C8A, CES1, CIRBP, COG1, CTSF, DAZAP2, DBF4B, DCXR, DNAJB5, EEF1A1, EIF2AK4, EIF4A2, FBOX31, FEZ1 FNDC1, FUS, GNB2, GNB2L1, GOLGB1, GPRC5C, H2AFY2, HEMK1, HN1, HP, KLHDC2, KREMEN1, LSAMP, MACF1, MAP1B, MAP1S, MAP2K5, MARCKSL1, MT-CO1, MT-ND1, MT-ND4L, NEIL1, NR2F6, PCDH8, PCDL, PEBP1, PPIE, PSAP, PSMB1, PSMB4, RBP4, RNF168, RNF187, RTN1, SARAF, 27A5, SLC2A1, SLC35A2, TF, TIMM50, TMX2, TPST2, TUBB, TUBB2A, VTN, ZDHHC6

a Names of proteins involved in interactions verified by coimmunoprecipitation assay are underlined.

b Host proteins that are unable to interact with the ORF1 polypeptide of g-1 HEV.

**TABLE 3:** Primary interaction (H_HEV_) partners that interact with multiple viral proteins

Host protein	HEV proteins	Biological process(es)
eIF3A	RdRp, ORF4	Formation of cytoplasmic translation initiation complex
eIF4A2	RdRp, V	Regulation of translation initiation
eEF1A1	RdRp, PCP	Translation elongation factor activity
ACTG1	RdRp, Met	Structural constituent of cytoskeleton
BTBD6	RdRp, V	Proteasome-mediated ubiquitin-dependent protein catabolic process
HP	RdRp, ORF3, ORF4	Acute-phase response
FGA	ORF3, ORF4	Acute-phase response, blood coagulation
FGB	ORF3, ORF4	Blood coagulation
TF	RdRp, ORF3, Hel	Positive regulation of receptor-mediated endocytosis, cellular iron ion homeostasis
VTN	RdRp, ORF3	Positive regulation of receptor-mediated endocytosis, regulation of complement activation
APOH	ORF3, ORF4	Blood coagulation
C3	RdRp, V	Complement activation
AZGP1	ORF4, X	Immune response
ALB	RdRp, V	Blood coagulation, negative regulation of apoptotic process
APCS	ORF3, Met	Acute-phase response
MT-CO1	RdRp, V	Oxidation-reduction process
MT-CO2	ORF3, ORF4	Oxidation-reduction process
ALDOB	RdRp, ORF3, ORF4, X, Met	Glucose metabolic process
ACY1	ORF3, Met, Hel	Cellular amino acid metabolic process
RBP4	X, Met	Retinol metabolic process, glucose homeostasis
HEMK1	Met, Hel	DNA methylation, protein methylation
DBF4B	ORF3, Met	Positive regulation of cell proliferation
C1orf61	ORF3, X	Unknown

**FIG 2:**
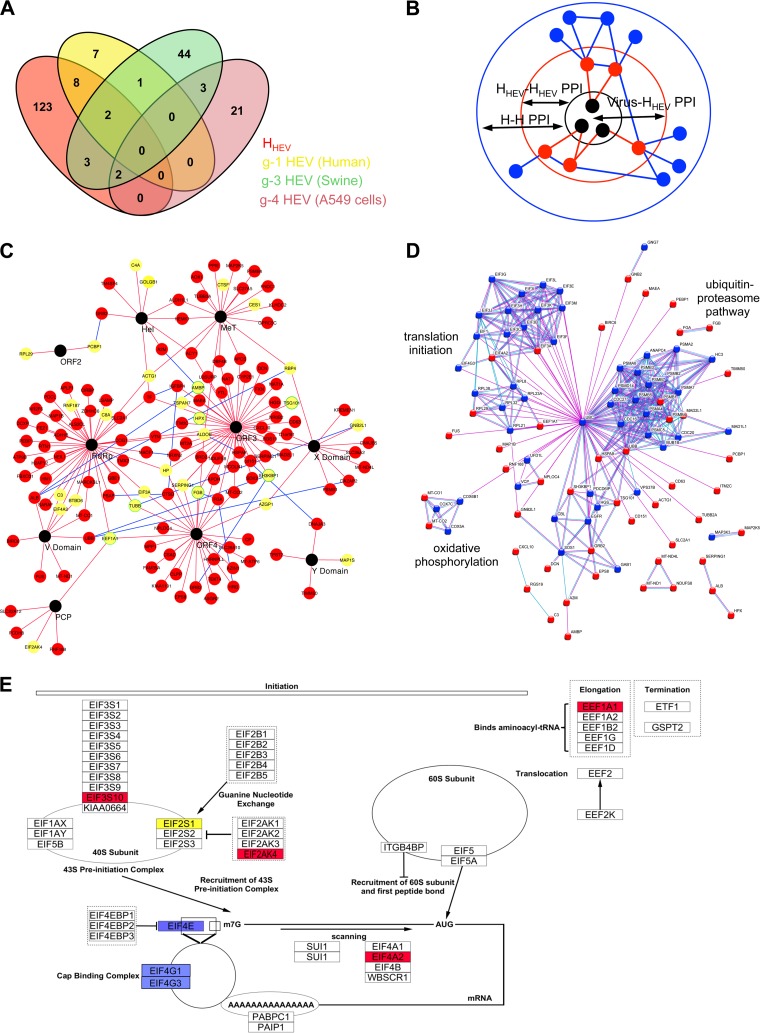
Construction and analysis of the HEV-host PPI network. (A) Venn diagram comparing the data from the primary interaction partners (H_HEV_) with the available proteomic data sets. Red, yellow, green, and pink indicate H_HEV_ and differentially regulated proteins in g-1 HEV-infected humans, g-3 HEV-infected swine, and a g-4 HEV-infected A549 cell line, respectively. (B) Schematic of the network analysis. Black, red, and blue nodes indicate viral protein and primary and secondary interaction partners, respectively. Red edge, virus-human (primary interaction partners) protein interaction (Virus-H_HEV_). Blue edge, all human-human (H-H) protein-protein interactions, including interactions among primary interaction partners (H_HEV_-H_HEV_) and experimentally validated interactions among all human proteins (H-H). (C) Virus-(H_HEV_-H_HEV_) PPI network. Yellow node, validated by CoIP. Yellow node with green border, virus-H_HEV_ interactions validated by CoIP, published data. (D) Analysis of the H_HEV_-H data set using STRING database. (E) The translation factor pathway, imported from WikiPathways (ID: WP107). HEV primary and secondary interaction partners are shown in red and blue, respectively. eIF2S1, the target of eIF2AK4, is indicated in yellow.

In order to further evaluate the specificity of the data set, we tested the ability of g-1 HEV-interacting host proteins to associate with their corresponding g-3 viral proteins. Among the g-1 HEV-interacting host proteins, 70% (78/111) could also interact with g-3 proteins (Table 2; see also Table S2B). Next, coimmunoprecipitation (CoIP) was performed for a number of interactions to confirm the data obtained by the Y2H assay. Flag- or hemagglutinin (HA)-tagged constructs of different viral proteins were transfected into Huh7 cells, and cell lysate was prepared in CoIP buffer. An aliquot of the cell lysate was immunoblotted with GAPDH (glyceraldehyde-3-phosphate dehydrogenase) antibody to ensure that equal amounts of protein were used in all CoIPs (Fig. 1B and C, upper panels). After CoIP, aliquots of the samples were immunoblotted with anti-Flag or anti-HA antibody to confirm the expression of corresponding Flag-tagged or HA-tagged viral proteins (Fig. 1B and C, lower panels). Note that Met-Flag showed a weak signal compared to other proteins. Therefore, a longer exposure of the cropped blot showing the relevant band is presented (Fig. 1B, right panel). Immunoblotting of aliquots of the CoIP samples using antibodies against different host proteins confirmed 23 interactions in Y2H (Fig. 1D). Interestingly, three additional interactions were observed (ORF4 interaction with CES1 and C8 and RdRP interaction with golgin B1) which were not detected by the Y2H assay (Fig. 1D, denoted by a single asterisk [*]). Further, using Flag- or HA-tagged expression clones of the host proteins, 9 interactions were confirmed among the 12 tested (Fig. S1A to L). Thus, 32 (~91%) of 35 interactions were independently verified to be positive by CoIP in this study (Fig. 2C [yellow nodes] and Table 2 [underlined proteins]). A total of 9 interactions had been validated earlier (13, 22) (Fig. 2C [yellow nodes with green border]).

10.1128/mSystems.00135-17.2 FIG S1  Confirmation of Y2H assay data by CoIP. (A to L) CoIP of the indicated host proteins by the corresponding viral proteins (bottom panel in each figure), revealed by Western blotting. WCE, whole-cell extract; IP, immunoprecipitation. WCE represents 25% of total protein used in IP. Download FIG S1, PDF file, 0.2 MB. Copyright © 2018 Subramani et al. 2018 Subramani et al. This content is distributed under the terms of the Creative Commons Attribution 4.0 International license.

Differential protein profiles of expression that occurred during HEV infection have been reported for g-1, g-3, and g-4 viruses (24–26). Comparison of the H_HEV_ data set with the g-1, g-3, and g-4 virus data sets revealed that 10/18 (g-1), 7/55 (g − 3), and 2/26 (g − 4) differentially expressed proteins also interact with a viral protein, which might account for their differential expression results (Fig. 2A).

### Construction and analysis of the HEV-human PPIN.

A PPI network (PPIN) was constructed from the H_HEV_ data set, using “cytoscape” (29), following the schematic shown in Fig. 2B, in which black, red, and blue nodes denote virus, human primary (H_HEV_), and human secondary (H) interacting proteins, respectively. The primary interaction network revealed that 148 nodes representing the virus-H_HEV_-H_HEV_ population were connected through 188 edges (Fig. 2C). Topological analyses revealed its clustering coefficient, centralization, and characteristic path length values to be 0.021, 0.279, and 3.6, respectively. Comparison of these parameters to that of the known human protein interaction network (constructed using the data from the HPRD [human protein reference database]) (30) (number of nodes, 9,454; number of edges, 36,870; clustering coefficient value, 0.013; centralization value, 0.028; characteristic path length, 4.2) indicated that the virus-H_HEV_ network could potentially be significant. Gene ontology (GO) analysis of the data set described above using the BiNGO (biological networks gene ontology) app (31) in cytoscape indicated the enrichment of proteins involved in stress and immune responses, protein and iron metabolism, oxidative phosphorylation, the ubiquitin-proteasome pathway, and cellular translation (Table S5A). Secondary interaction partners of the virus-H_HEV_ population were identified from the human protein interaction network, and the virus-H_HEV_-H (virus-host primary and secondary interactions) PPI network was generated (1,226 nodes and 5,697 edges; Fig. S2). The results of BiNGO analysis of the data set described above were in agreement with the results of the H_HEV_ data set analysis (Table S5B). The functional significance of the H_HEV_ population was also analyzed in the STRING database (32) with the following parameters: data source, experimentally validated data and curated databases; confidence level, highest (0.9); maximum number of interaction partners, 50 (H_50_). STRING results were mostly similar to the BiNGO results (Table S5C). Notably, factors involved in cellular translation and oxidative phosphorylation and proteasome components were represented in distinct clusters (Fig. 2D). Further analyses revealed that viral proteins interact with host factors involved in three different stages of translation: preinitiation complex assembly, scanning, and elongation (red nodes, Fig. 2E).

10.1128/mSystems.00135-17.3 FIG S2  Virus-H_HEV_-H PPI network. Black, red, and blue nodes represent viral factors, primary host interaction partners, and secondary host interaction partners, respectively. Download FIG S2, PDF file, 0.6 MB. Copyright © 2018 Subramani et al. 2018 Subramani et al. This content is distributed under the terms of the Creative Commons Attribution 4.0 International license.

10.1128/mSystems.00135-17.10 TABLE S5  Functional analysis of the virus-H_HEV_ and virus-H_HEV_-H data sets in BiNGO and H_HEV_-H (50) data set in STRING. Download TABLE S5, XLSX file, 0.4 MB. Copyright © 2018 Subramani et al. 2018 Subramani et al. This content is distributed under the terms of the Creative Commons Attribution 4.0 International license.

### Several host translation regulatory factors are incorporated into a multiprotein complex composed of viral RdRp, X, helicase, PCP, V, methyltransferase, and ORF4.

**FIG 3:**
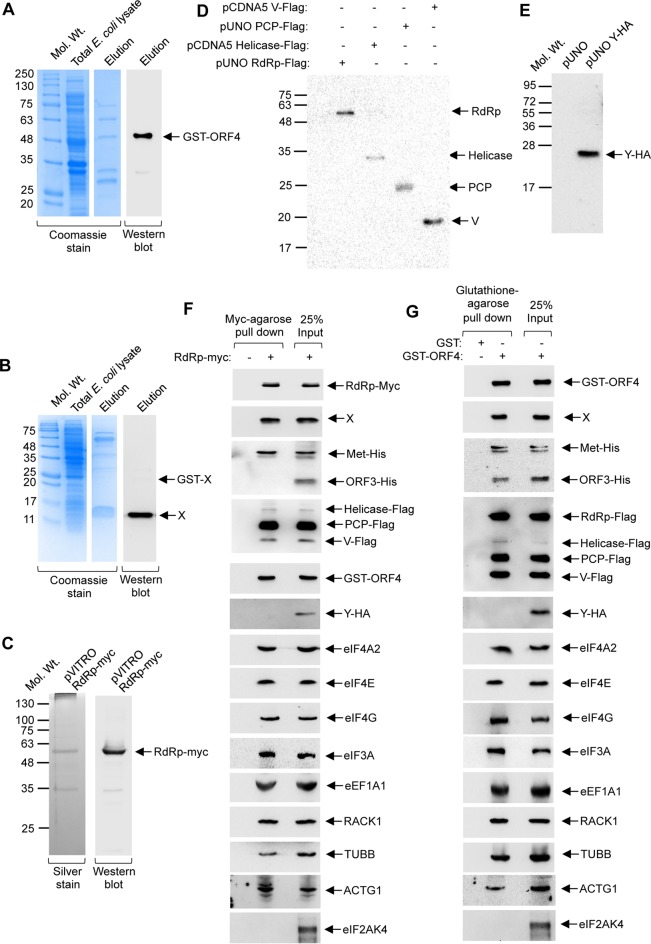
Several host translation factors are present in the protein complex consisting of the viral RdRp, X, helicase, methyltransferase, PCP, V domain, and ORF4. (A) Coomassie (left) and Western blot (using ORF4 antibody; right) analysis of GST-ORF4 protein, purified from *E. coli*. (B) Coomassie (left) and Western blot (using X antibody; right) analysis of the X protein, purified from *E. coli*. (C) Silver stain (left) and Western blot (using myc antibody, right) analysis of RdRp-myc protein, purified by myc-agarose beads, from Huh7 cells overexpressing the protein. (D) Western blot (using Flag antibody) analysis of RdRp, helicase, PCP, and V proteins, purified by Flag-agarose beads, from Huh7 cells overexpressing them. (E) Western blot analysis (using HA antibody) of Y purified by HA-agarose beads, from Huh7 cells overexpressing them. (F) Myc-agarose pulldown assay using RdRp-Myc protein, followed by Western blot analysis of viral proteins and host factors using the indicated antibodies. (G) GST pulldown assay using GST-ORF4 protein, followed by Western blot analysis of virus and host factors using the indicated antibodies.

Taking cues from the analysis described above, a pulldown assay was conducted to verify whether the host translation factors are associated with the viral RdRp-mediated translation/replication complex *in vitro*. Individual viral proteins were affinity purified from *Escherichia coli* cells (X and ORF4 [this study, Fig. 3A and B] and methyltransferase and ORF3 [as described previously [33]) or Huh7 cells (RdRp-Flag, RdRp-myc, helicase-Flag, PCP-Flag, V-Flag, and Y-HA [Fig. 3C to E]). Huh7 cells expressing N-terminal myc-tagged RdRp were immobilized on myc-agarose beads, followed by incubation with purified viral proteins and Huh7 human hepatoma cell extract. Unbound proteins were washed out, and RdRp-myc associated proteins were identified by Western blotting (Fig. 3F). Huh7 cells lacking RdRp-myc served as the negative control. A total of 25% of the sample used for binding was run in parallel to monitor the binding efficiency.

In agreement with our previous report (13), X, helicase, and ORF4 were present in the RdRp protein complex (Fig. 3F). Methyltransferase, V, and PCP were also present in the same complex, whereas ORF3 and the Y domain were absent (Fig. 3F). Among the directly interacting host translation factors (H_HEV_) probed, eIF4A2, RACK1 (GNB2L1), eIF3A, and eEF1A1 were present, whereas eIF2AK4/GCN2 was not part of the complex. TUBB and ACTG1 were also probed as they are direct interaction partners of RdRp, and our earlier study suggested that they could be present in the viral translation/replication complex. Both were detected in the Western blot (Fig. 3F).

In order to verify whether the factors identified by RdRp-myc pull down indeed remained in one complex, we repeated the assay using glutathione *S*-transferase (GST) (Fig. S3A) or GST-ORF4 as the bait protein. Instead of RdRp-Myc, purified RdRp-Flag protein was used. A similar pattern was observed with the exception that the ORF3 protein was also pulled down (Fig. 3G). This was attributed to the fact that ORF3 is a direct interaction partner of ORF4 (13).

10.1128/mSystems.00135-17.4 FIG S3  Evaluation of the silencing efficiency and cytotoxicity of siRNAs and shRNA. (A) Coomassie stain of the GST protein, produced in *E. coli*. (B to G) Western blot analysis of Huh7 cells expressing scrambled siRNA or eIF4A2 siRNA (B), eIF3A siRNA (C), RACK1 siRNA (D), eIF2AK4 siRNA (E), ACTG-1 siRNA (F), or enhanced green fluorescent protein (EGFP) or eEF1A1 shRNA (G). (H) (Upper panel) Western blot showing the level of nascent protein synthesized in Huh7 cells during 2-h labeling period. Cells were transfected with the indicated siRNAs 72 h prior to labeling. (Lower panel) Coomassie staining of Western blot membrane shown in upper panel. (I) Estimation of viability of Huh7 cells transfected with the indicated siRNA/shRNA for 72 h. In the case of siRNA-transfected samples, the value of the scrambled siRNA (scr) transfected sample was considered to be 100% and all other values were calculated with reference to that. In the case of eEF1A1 transfected samples, the value of the EGFP shRNA-transfected sample was considered to be 100% and all other values were calculated with reference to that. Values are means ± SEM of data from triplicate samples. (J) Fluorescence-activated cell sorter (FACS) plot of asynchronous Huh7 cells maintained in DMEM, supplemented with 10% FCS. (K) FACS plot of G0/G_1_-arrested Huh7 cells maintained in DMEM, supplemented with 10% FCS and 2 mM thymidine (double thymidine block). (L) QRT-PCR of HEV sense RNA level in Huh7 cells transfected with *in vitro*-synthesized capped g-1 HEV RNA, maintained in DMEM supplemented with 10% FCS (asynchronous) or DMEM supplemented with 10% FCS and 2 mM thymidine (G0/G_1_ arrested). Relative RNA levels (normalized to that of GAPDH) are represented as means ± SEM. (M) Measurement of *Renilla* luciferase activity in Huh7 cells electroporated with *in vitro*-synthesized capped p6 HEV-Luc RNA. Cells were maintained in DMEM supplemented with 10% FCS (asynchronous) or DMEM supplemented with 10% FCS and 2 mM thymidine (G0/G_1_ arrested). Levels of viability of the same cells were measured by MTT assay. *Renilla* luciferase values were normalized to that of the former and are represented as means ± SEM. Download FIG S3, PDF file, 2.2 MB. Copyright © 2018 Subramani et al. 2018 Subramani et al. This content is distributed under the terms of the Creative Commons Attribution 4.0 International license.

### ORF1 polypeptide associates with ORF4 and displays a host interaction profile highly similar to those of its individual domains.

**FIG 4:**
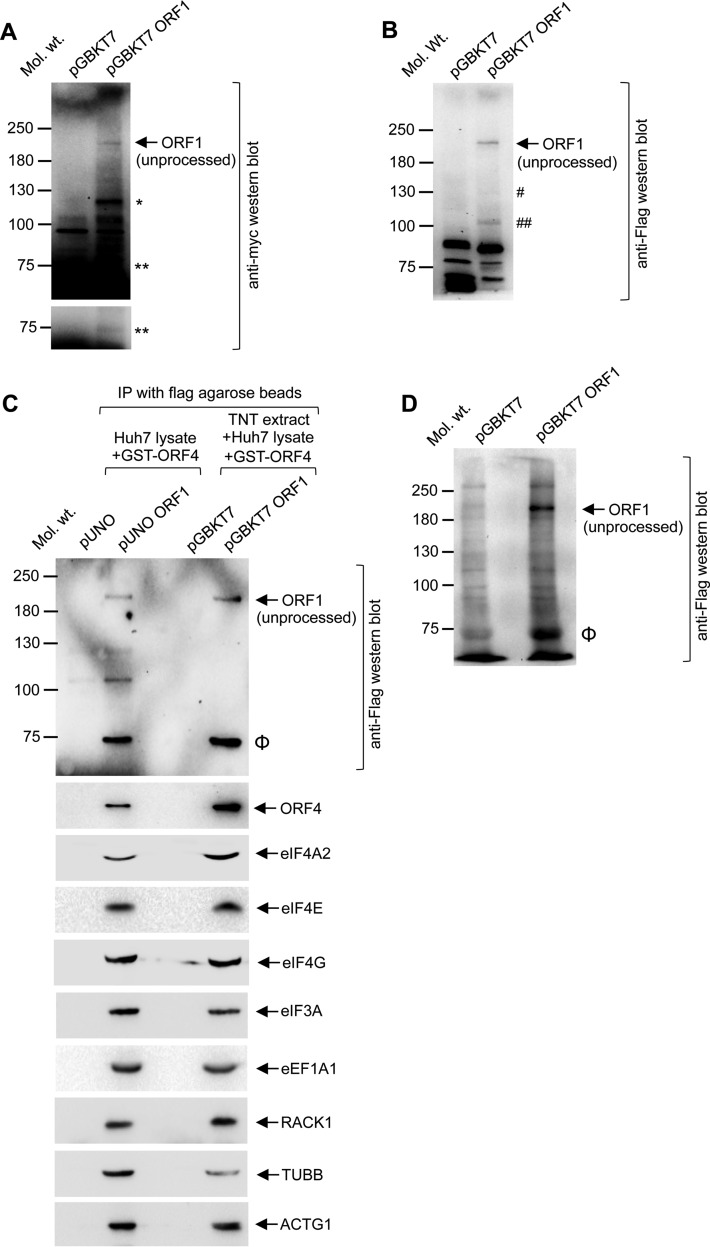
ORF1 polypeptide associates with host translation factors and assembles a multiprotein complex. (A) Western blot analysis of pGBKT7 and pGBKT7 ORF1 expressing Y2H gold whole-cell extract using anti-Myc antibody (upper panel). A shorter-exposure image is shown to clarify the band at the 75-kDa position (lower panel). Single asterisks (*) and double asterisks (**) denote processed ORF1 fragments. (B) Western blot analysis of pGBKT7 and pGBKT7 ORF1 expressing Y2H gold whole-cell extract using anti-Flag antibody. Single crosshatch symbols (#) and double crosshatch symbols (##) denote processed ORF1 fragments. (C) Coimmunoprecipitation of ORF1-Flag-associated proteins in Huh7 cells using Flag agarose beads (pUNO and pUNO ORF1, lanes 1 and 2 from left). GST-ORF4 was added to the lysate prior to addition of Flag agarose beads. Aliquots of IP samples were subjected to Western blotting using the indicated antibodies. Data represent results of a Flag-agarose pulldown assay of a mixture of *in vitro*-translated ORF1-Flag, GST-ORF4, and Huh7 whole-cell extracts (pGBKT7 and pGBKT7 ORF1, lanes 3 and 4 from left). Aliquots of eluates were subjected to Western blotting using the indicated antibodies. An uppercase Greek phi (Φ) denotes a processed ORF1 fragment. (D) Western blot analysis of pGBKT7 and pGBKT7 ORF1 synthesized by TNT using anti-Flag antibody. An uppercase Greek phi (Φ) denotes the processed ORF1 fragment.

In contrast to many viruses, no clear evidence exists regarding the processing of HEV ORF1 polypeptide into individual domains although distinct functional domains have been predicted. On the basis of previously reported studies (summarized in the introduction), it may be possible either that ORF1 is processed into distinct functional domains during the course of natural infection (by HEV protease or host proteases or a combination of the two), which no one has been able to monitor until now, or that ORF1 processing is an inefficient process and the unprocessed polypeptide is functionally active. In any case, the HEV-host PPI network established by us would be useful only if such interactions were relevant during the course of HEV infection. Therefore, we next tested the validity of the HEV-host PPI network in the context of ORF1 polypeptide. A ORF1 region with an N-terminal Myc tag and a C-terminal Flag tag was cloned into binding domain (BD) vector pGBKT7, followed by verification of ORF1 expression in Y2H gold cells using both Myc and Flag antibodies. A band corresponding to the size of unprocessed ORF1 along with BD (1,841 aa) was detected at ~220 kDa, with both Myc and Flag antibodies (Fig. 4A and B). Interestingly, two additional bands corresponding to ~120 kDa and ~75 kDa were detected using Myc antibody (Fig. 4A, marked with single asterisks [*] and double asterisks [**], respectively) and two bands corresponding to ~130 kDa and ~100 kDa were detected using Flag antibody (Fig. 4B, marked with single crosshatch symbols [#] and double crosshatch symbols [##], respectively). These results demonstrated that although BD-fused ORF1 is present as unprocessed polypeptide in Y2H gold cells, a fraction of it is likely processed into smaller fragments. As unprocessed ORF1 was clearly detectable, we proceeded with the Y2H assay to evaluate the ability of ORF1 polypeptide to associate with host proteins that interacted with its individual domains. Of the 84 unique host proteins that interacted with different ORF1 domains (note that 14 host proteins interact with two or more ORF1 domains), 79 could associate with ORF1 polypeptide (Table 2; see also Table S2C). A total of 5 proteins did not show any interaction with ORF1 (Table 2, proteins with footnote *b*). ORF4 also interacted with ORF1 polypeptide (Table S2C).

Next, we tested whether ORF1 polypeptide could assemble the protein complex in a manner similar to that exhibited by its individual domains. A C-terminal Flag-tagged ORF1 expression plasmid was transfected into Huh7 cells. At 48 h posttransfection, whole-cell lysate was prepared from 2 million cells and 200 ng purified GST-ORF4 protein was mixed with it, followed by IP performed using Flag-agarose beads. Western blot analysis of IP samples using anti-Flag antibody revealed an ~200-kDa band, corresponding to the size of unprocessed ORF1 polypeptide, which was not present in the IP sample that lacked ORF1 (Fig. 4C, pUNO). A smaller band corresponding to ~75 kDa was also detected, suggesting processing of a fraction of ORF1 polypeptide (Fig. 4C, marked by an uppercase Greek phi [Φ]). Note that a band that was present above 100 kDa was not considered specific to ORF1, as it was also detected in the pUNO IP, though as a weaker signal. In order to further confirm the specificity of the band corresponding to unprocessed ORF1 polypeptide, an *in vitro* coupled transcription-translation (TNT) assay was performed to produce a C-terminal Flag-tagged ORF1 polypeptide using the pGBKT7 ORF1 plasmid, in which ORF1-Flag is under the control of the T7 promoter. Note that T7-expressed ORF1-Flag is not fused to the GAL4-BD; hence, its size should be similar to that of the unprocessed ORF1 polypeptide produced in Huh7 cells, unless the latter is posttranslationally modified in a manner not supported by the rabbit reticulocyte lysate. Western blot analysis of the pGBKT7 empty vector and pGBKT7 ORF1 TNT samples revealed a pattern similar to that observed in Huh7 cells (Fig. 4D), confirming that the ~200-kDa band corresponds to unprocessed ORF1 polypeptide and that the smaller ~75-kDa band is a product derived from ORF1. Next, TNT-expressed ORF1 and control lysates were mixed with Huh7 cell lysate (from 2 million cells) and 200 ng GST-ORF4 protein, followed by IP with Flag-agarose beads. Anti-Flag Western blot analysis of an aliquot of the sample revealed a pattern similar to that observed in the IP sample of ORF1-expressing Huh7 cells (Fig. 4C), confirming that the ~200-kDa band detected in both of the samples corresponded to the unprocessed ORF1 polypeptide. Aliquots of both Huh7 and TNT-expressed ORF1 IP samples were subjected to Western blotting using various antibodies to identify proteins associated with them. ORF4 and host factors eIF4A2, eIF4E, eIF4G, eIF3A, eEF1A1, RACK1, TUBB, and ACTG1 were coprecipitated by ORF1 in both samples (Fig. 4C). Although it is not possible to conclude from the CoIP assay whether all the factors listed above are associated with unprocessed or processed ORF1 (the 75-kDa protein is likely to represent a C-terminal fragment of ORF1, containing at least helicase and RdRp domains), taken together with the Y2H data, these results suggest that unprocessed ORF1 polypeptide displays an interaction profile similar to those of its individual domains and that a fraction of ORF1 is processed into smaller fragments, both *in vivo* and *in vitro*. Thus, the host translation factors identified in our screening may play a functional role in viral translation/replication, irrespective of the extent of ORF1 polypeptide processing.

### Host eIF4A2, eIF3A, eEF1A1, and RACK1 are essential for HEV translation/replication.

**FIG 5:**
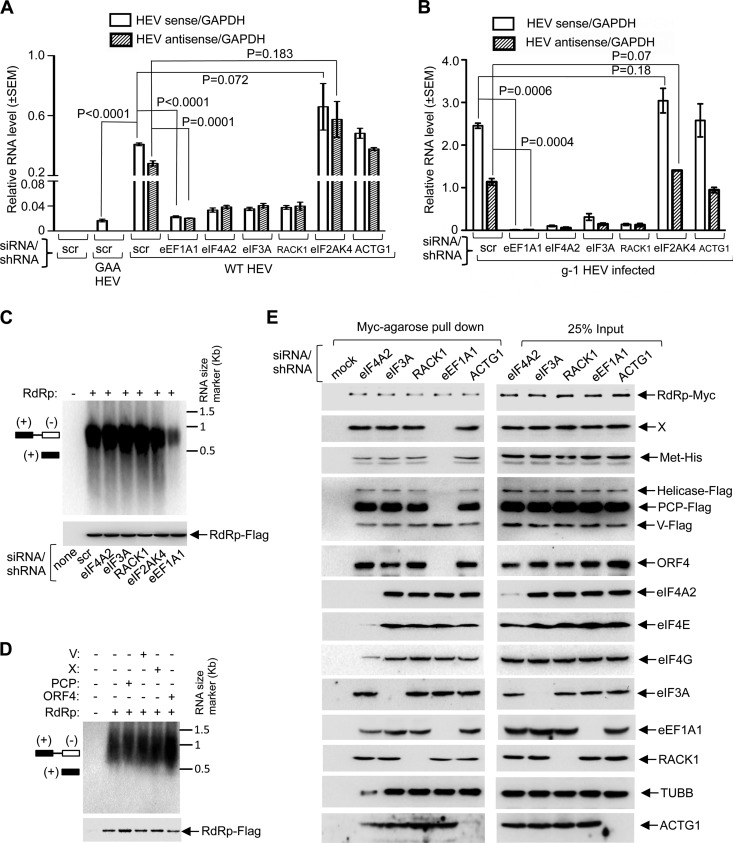
Essential role of host translation regulatory factors in the assembly of viral translation/replication complex. (A) QRT-PCR of sense and antisense RNA of mock, WT HEV, and GAA HEV infection in Huh7 cells expressing various siRNAs/shRNA, as indicated. Relative RNA levels (normalized to that of GAPDH) are represented as means ± SEM. (B) QRT-PCR of sense and antisense RNA of g-1 HEV-infected ORF4-Huh7 cells expressing various siRNAs/shRNA, as indicated. Relative RNA levels (normalized to that of GAPDH) are represented as means ± SEM. (C) (Upper panel) HEV RdRp assay using the indicated siRNA/shRNAs and RdRp-Flag-transfected cells. (Lower panel) Western blot analysis of aliquots of RdRp protein used in the assay, performed using Flag antibody. (D) (Upper panel) HEV RdRp assay using affinity purified RdRp-Flag and the indicated viral proteins from Huh7 cells. (Lower panel) Western blot analysis of aliquots of RdRp protein used in the assay, performed using Flag antibody. (E) Myc-agarose pulldown assay of the indicated siRNAs/shRNA and RdRp-myc-transfected Huh7 cells, followed by Western blot analysis of various viral and host factors, performed using the indicated antibodies.

In order to understand the functional significance of host translation factors in HEV replication, we measured viral sense and antisense RNA levels in Huh7 cells depleted of those factors. eIF4A2, eIF3A, RACK1, eIF2AK4, and ACTG1 expression was altered using specific small interfering RNAs (siRNAs) against them (Fig. S3B to F). eEF1A1 expression was knocked down using short hairpin RNA (shRNA), as previously described (13) (Fig. S3G). Cellular GAPDH protein levels were measured in aliquots of the sample described above to ensure equal loading of the samples. No significant change in the GAPDH protein level was detected in siRNA-treated and shRNA-treated cells (Fig. S3B to G, lower panels). In order to verify whether knockdown of the translation regulatory factors resulted in a global reduction in the level of protein translation, newly synthesized proteins were incubated with l-azidohomoalanine (AHA) in siRNA- and shRNA-transfected Huh7 cells followed by measurement of AHA incorporation by a chemiluminescence-based technique (see Materials and Methods). A moderate reduction in the level of translation was observed in the samples treated with siRNAs against RACK1 and GCN2 and with shRNA against eEF1A1 (Fig. S3H, upper panel). The same blot was subjected to Coomassie staining to monitor loading of the sample (Fig. S3H, lower panel). To check for possible cytotoxicity due to the lack of translation factors, the levels of viability of the siRNA- and shRNA-transfected Huh7 cells were measured by a tetrazolium salt-based assay. No cytotoxicity was observed for any of the siRNA/shRNA-treated cells (Fig. S3I). Next, *in vitro*-transcribed capped RNAs of full-length g-1 HEV (wild-type [WT] HEV) or its replication-defective mutant (GAA HEV) (13) were transfected into Huh7 cells. siRNAs and shRNAs were transfected into these cells twice, as described in Materials and Methods. At 72 h posttransfection, total RNA was isolated and quantitative reverse transcription-PCR (QRT-PCR) was performed (Fig. 5A). Levels of viral sense and antisense RNA were significantly reduced in the eIF4A2, eIF3A, and RACK1 siRNA-transfected cells, whereas eIF2AK4 siRNA-transfected cells showed a marginal increase in both sense and antisense RNA levels, though the increase was not statistically significant (Fig. 5A). ACTG1 depletion had no effect on viral RNA levels (Fig. 5A). As observed earlier (13), viral sense and antisense RNA levels were significantly reduced in cells lacking eEF1A1 (Fig. 5A). Note that both the sense and antisense RNA levels were normalized to the GAPDH RNA level and are represented as relative RNA levels to rule out experimental artifacts.

Next, an ORF4-Huh7 cell-based infection model of g-1 HEV (34) was used to verify the data present above. ORF4-Huh7 cells were infected with a g-1 HEV clinical isolate, following a previously optimized protocol (34). At 72 h postinfection, cells were transfected twice with siRNAs and shRNA against different host factors. At 72 h posttransfection, total RNA was isolated followed by measurement of HEV sense and antisense RNA levels (normalized to the GAPDH RNA level) using QRT-PCR. In agreement with the HEV RNA transfection data, eEF1A1, eIF4A2, eIF3A, and RACK1 depletion significantly inhibited viral replication, whereas eIF2AK4 and ACTG1 knockdown had no effect (Fig. 5B). To assess the possibility that the observed reduction in viral sense and antisense RNA levels could be attributed to an indirect effect of the siRNAs/shRNA on cell cycle progression, we evaluated the effect of cell cycle inhibition on HEV replication. A double thymidine block protocol was followed to arrest the Huh7 cells at the G0/G_1_ phase of the cell cycle, as reported previously (35). DNA content measurement revealed that thymidine-blocked Huh7 cells contained 92.8%, 4.48%, and 0.44% populations in the G0/G_1_, S, and G_2_/M phases, compared to 64.1%, 5.72%, and 26.3% in the asynchronous Huh7 cells, respectively, indicating that the protocol described above could be followed to inhibit cell cycle in Huh7 cells (Fig. S3J and K). Next, g-1 HEV RNA-transfected Huh7 cells were subjected to a double thymidine block for 44 h after 5 days of transfection, followed by further incubation in thymidine for 24 h. Aliquots of the cells were used for measurement of HEV sense and GAPDH RNA levels by QRT-PCR and analysis of DNA content by propidium iodide staining. A significant increase in the viral RNA level was observed in G0/G_1_-arrested cells, indicating increased viral replication (Fig. S3L). A similar increase in viral replication was observed in g-3 HEV replicon (P6 HEV-Luc)-expressing Huh7 cells that had been treated with thymidine as described above for g-1 HEV (Fig. S3M).

Next, an RdRp assay was conducted to assess whether the host translation regulatory factors acted on viral RdRp activity. At 48 h posttransfection of the different siRNAs and shRNA into Huh7 cells, the same cells were transfected with a plasmid encoding the Flag-tagged g-1 HEV RdRP, followed by 48 h of incubation in complete medium. RdRp was partially purified from the cells described above, and equal amounts of protein were incubated with a HEV-specific single-stranded RNA (ssRNA) template in the presence of DIG-11-UTP (digoxigenin-11-UTP). DIG incorporation into the nascent strands as a result of the RdRp activity was revealed by chemiluminescence using the antibody against it, as described in Materials and Methods and reported previously (13, 36). Except for eEF1A1 (which was previously reported [13]), no host factor had any effect on RdRp activity (Fig. 5C). Next, we evaluated the effect of other viral factors on RdRp activity. Approximately equal amounts of partially purified RdRp and ORF4, PCP, X, or V domain proteins (in different combinations, as indicated in Fig. 5D) were incubated with a HEV-specific ssRNA template in the presence of DIG-11-UTP, followed by measurement of the incorporated DIG signal, as mentioned above. Only ORF4 enhanced the RdRp activity, in agreement with our earlier report (13). No other viral protein had any effect on the activity of viral RdRp *in vitro* (Fig. 5D). Next, an RdRp-myc pulldown assay was conducted to evaluate the effect of a lack of translational regulatory factors on the assembly of viral translation/replication complex. Lack of eIF4A2 abolished the binding of eIF4E and significantly reduced the binding of eIF4G, TUBB, and ACTG1, whereas lack of eIF3A, RACK1, and ACTG1 had no effect on binding of other factors probed (Fig. 5E). Importantly, lack of eEF1A1 had the most profound effect on the protein complex, as it abolished the binding of X, methyltransferase, helicase, PCP, and ORF4 to the RdRp complex (Fig. 5E). Thus, recruitment of the majority of the viral factors to the translation/replication complex is dependent on eEF1A1.

Further, an immunofluorescence assay was performed to visualize whether host translation factors colocalized with viral replication complex. A double-stranded RNA (dsRNA)-specific antibody was used to visualize viral replication. Note that earlier studies had demonstrated the reliability and specificity of dsRNA antibody for detecting replicating viral dsRNA (37). Huh7 cells transfected with *in vitro*-synthesized capped genomic RNA of HEV were processed for immunofluorescence assay, 5 days posttransfection, using antibodies against dsRNA, X, eIF3A, eEF1A1, eIF4A2, and RACK1. dsRNA (red color) colocalized with both eEF1A1 (green color) and eIF3A (green color) as evident from the yellow color of red and green superimposed fields (Fig. S4, white arrows). Yellow arrows indicate cells lacking dsRNA staining in the same field, confirming the specificity of the signal (Fig. S4). Since mouse monoclonal antibodies against eIF4A2 and RACK1 were functional for immunofluorescence staining and the dsRNA antibody was also of similar origin, it was impossible to perform colocalization studies using those antibodies. However, as our previous experiments demonstrated the viral X protein to be a part of the translation/replication complex and as the rabbit polyclonal antibody against X was functional in immunofluorescence assays, we performed studies of colocalization between X and eIF4A2 and between X and RACK1. Both eIF4A2 and RACK1 colocalized with X (white arrow, Fig. S4).

10.1128/mSystems.00135-17.5 FIG S4  Immunofluorescence assay illustrating colocalization of dsRNA and X protein with host translation regulatory factors. Huh-7 cells were transfected with *in vitro*-synthesized wild-type g-1 HEV genomic RNA and stained with indicated antibodies. Nuclei were stained with DAPI (blue); X, eIF3A, and eEF1A1 were stained with Alexa Fluor 488 (green); and dsRNA, RACK1, and eIF4A2 were stained with Alexa Fluor 594 (red). Colocalization is visible as yellow signal in superimposed images, denoted by white arrows. Yellow arrows denote untransfected cells. Scale, 20 μm. Download FIG S4, PDF file, 1.4 MB. Copyright © 2018 Subramani et al. 2018 Subramani et al. This content is distributed under the terms of the Creative Commons Attribution 4.0 International license.

## DISCUSSION

PPIs are essential structural and communication components of an organism. Here, we constructed the first comprehensive PPI network of HEV and its human host and attempted to establish the functional significance of those interactions during the course of viral infection. A stringent procedure was followed to rule out false positives (38, 39) as follows. (i) The Y2H gold strain, which contains *AUR1-C* (which encodes a mutant inositol phosphoryl-ceramide synthase that confers resistance to the antibiotic aureobasidin A) reporter, in addition to *ADE1*, *HIS3*, and *MEL1*, was used for the screening (40). (ii) Only the interactions that activated all four reporters in the screening as well as in the retransformation assay were considered positive. (iii) Only the clones containing coding sequence in frame with the BD were manually selected as bona fide interaction partners. (iv) The g-1 HEV-host PPI profile was verified against respective g-3 HEV proteins. In summary, the data obtained in our study are likely to be of very high confidence for further analyses as they were selected through rigorous scrutiny. Though the interaction partners of some HEV proteins have been reported previously (22, 41), this is the first report of identification of the host interactome of all HEV proteins. Further, inclusion of a brain library in the screening identified several new interaction partners which were not obtained in the earlier studies, as they screened only the liver library.

A few studies on ORF1 processing have indicated that there is some processing of ORF1, *in vitro* and *in vivo* (14, 15). Data obtained by us in the current study also support the latter observations, as there is partial processing of ORF1 in yeast cells, Huh7 cells, and rabbit reticulocyte lysate. Further, in our studies, a clear band corresponding to the size of unprocessed ORF1 polypeptide was detected in all systems, indicating that a fraction of ORF1 had remained unprocessed. Our original screening involving individual domains of ORF1 allowed us to explore the full potential of each distinct domain to associate with host proteins. Comparison of the abilities of the members of the repertoire of host proteins isolated in the original screening to associate with ORF1 polypeptide revealed that 94% of the host proteins (79 of 84 unique host proteins) could associate with it. Moreover, ORF4, which is an essential component of the g-1 HEV translation/replication complex, could also directly associate with the ORF1 polypeptide and with several host factors in the Y2H assay as well as the CoIP assay, suggesting that such protein-protein interactions could be relevant during the natural course of infection.

The HEV-human PPI data were analyzed *in silico* to obtain a virtual view of the impact of the data on different host processes. Host proteins known to be involved in the ubiquitin-proteasome pathway and cellular translation were among the top hits of the GO analysis. PSMB1 and PSMB4, essential components of the 20S proteasome, interact with the X and methyltransferase, respectively, and the ubiquitin protein interacts with the V domain. The importance of the ubiquitin-proteasome system in the host is highlighted by the fact that it represents the predominant quality control machinery of the cell and is also essential for the processing of major histocompatibility complex (MHC) class I peptides. Thus, modulation of the proteasome activity by multiple HEV proteins might have a broader consequence. Indeed, many viruses exploit the host ubiquitin-proteasome system for their benefit and HEV replication has been reported to require the host ubiquitin-proteasome system (27, 42). Among other processes, proteins involved in oxidative phosphorylation and components of the complement and coagulation pathways were identified as interaction partners of HEV proteins. C1-inhibitor (SERPING 1), an important factor involved in the activation of C1 complex, interacts with ORF4. C3, the core component of both the classical and alternative complement activation pathways, interacts with two viral proteins (RdRp and V domain). C8 interacts with RdRp, ORF4, and helicase, and C4a interacts with RdRp and helicase. Interestingly, reduced C3 levels were observed in the plasma of g-1 HEV patients (24). Investigation of the significance of the interactions among C3, RdRp, and V might uncover the mechanism underlying their reduced levels in the patient plasma. Among the hepatitis-causing viruses, HCV NS5A is known to inhibit C3 and C4 and HCV core inhibits C4 and C9 (43–45). HCV NS3/4A protease also cleaves C4γ (46). Further, the C2 mRNA level is reduced and C3 convertase activity is inhibited in HCV patients (47). Thus, complement inhibition appears to be an important strategy of both HCV and HEV.

Considering the importance of the cellular translation factors in viral translation/replication (1), we explored the significance of the interaction between the former and HEV proteins. Our report provides the first evidence of the presence of host translation factors in the HEV translation/replication complex and also demonstrates the essentiality of those factors for viral replication. By virtue of its ability to directly interact with eIF4A2, HEV RdRp recruits host eIF4E and eIF4G into the viral replication complex. Note that eIF4A, eIF4E, and eIF4G together form the eIF4F complex and that components of the eIF4F complex are attractive targets for the viruses to usurp the host translation machinery. The virion host shutoff (Vhs) protein of herpes simplex virus 1 (HSV-1) and UL69 of HCMV bind to eIF4A; VPg of caliciviruses and tobacco mosaic virus binds to eIF4E; and NS1 of influenza virus and ICP6 of HSV-1 bind to eIF4G (6, 48–52). Interestingly, an earlier study indicated the involvement of eIF4F complex in HEV replication (28).

The eIF3 complex is a key component of the eukaryotic translation machinery, which coordinates the interactions among ribosomes, eIF4F complex, and mRNA and plays an essential role in scanning of the AUG initiation codon. It consists of 13 subunits (eIF3A to eIF3M). eIF3 complex is an attractive target for most viruses, as modulation of its activity by viral proteins helps in virus-RNA translation while shutting down host protein synthesis. Many viral proteins directly interact with eIF3 subunits to modulate its activity. Notably, VPg of caliciviruses binds to eIF3, spike protein of severe acute respiratory syndrome coronavirus (SARS-CoV) and infectious bronchitis virus (IBV) binds to eIF3F, and matrix protein of rabies virus interacts with eIF3 complex and, in all cases, the binding facilitates viral mRNA translation and/or inhibits host cell translation (53–55). In the case of HEV, viral RdRp and ORF4 were found to interact with eIF3A and pulldown assays confirmed the presence of eIF3A in the viral translation/replication complex. Further, silencing of eIF3a significantly inhibited viral replication, underscoring the importance of eIF3A in the HEV life cycle.

Receptor-activated kinase 1 (RACK1/GNB2L1) is a WD40 beta-propeller protein that acts as an adaptor protein, interacting with a variety of signaling molecules (such as protein kinase C [PKC], SRC, and mitogen-activated protein kinase [MAPK]). It is also a component of the 40S subunit of the ribosome. It has been shown to couple signaling pathways to the translation machinery (56). RACK1 has been shown to control internal ribosome entry site (IRES)-mediated translation of many viruses such as HCV, cricket paralysis virus (CrPV), vesicular stomatitis virus (VSV), and feline herpesvirus (FHV) (57). RACK1 also associates with VP5 of infectious bursal disease virus, resulting in inhibition of apoptosis and increase of viral replication (58). In our study, an HEV macrodomain was found to interact with RACK1 and silencing of the latter significantly reduced viral replication. RACK1 was also detected in the viral translation/replication complex. It is possible that RACK1 recruits the corresponding interacting proteins such as PKC to the viral translation/replication complex, which in turn would influence the activity of other components of the complex, thereby promoting viral translation/replication. In summary, this report identifies and validates the importance of cellular translation regulatory factors in HEV translation/replication.

Among the host factors present in the viral translation/replication complex, eEF1A1 appears to be the most crucial one not only because its absence results in dissociation of five viral proteins (X, methyltransferase, helicase, PCP, and ORF4) from the multiprotein complex but also because its absence significantly reduces the activity of viral RdRp *in vitro*. Thus, eEF1A1 is essential for optimal RdRp activity and stabilization of the viral translation/replication complex.

The current report also provides new insights into the emerging issue related to molecular differences in the functional properties of the proteins encoded by the different HEV genotypes. Recently, ORF4 was shown to be crucial for replication of g-1 HEV and it was also observed that g-1 and g-3 viral proteins differ in their ability to interact with other viral proteins (13). In the context of virus-host PPI, our data reveal that 70% of host proteins are common between g-1 and g-3 HEV. Thus, the g-3 HEV interactome possibly contains additional host proteins. However, since all translation factors interact with both g-1 and g-3 HEV proteins, the results obtained in this study with respect to viral translation/replication are probably applicable to other HEV genotypes as well. Nevertheless, it would be interesting to identify the host-virus PPI network of g-3 HEV and compare the data to those from the g-1 HEV. Information obtained from such studies is likely to identify suitable targets for developing specific antivirals against HEV.

In conclusion, our report provides a vast resource of virus-host PPI data for exploring the impact of HEV infection on the host. It also provides the first evidence of the physical presence of cellular translation factors in the HEV translation/replication complex and demonstrates their functional importance in viral replication.

## MATERIALS AND METHODS

### Reagents and antibodies.

The Y2H gold strain, a Matchmaker Mate & Plate human liver and fetal brain cDNA library, X alpha galactosidase (α-Gal), and aureobasidin A were from Clontech (Mountain View, CA, USA). A TNT T7 kit and a CellTiter 96 non-radioactive cell proliferation assay kit were from Promega (Madison, WI, USA). Antibodies were obtained from following sources: from Santa Cruz Biotechnology (Dallas, TX), Flag, Myc (9E10), His, RPL29, C3, SERPING1, GCN2, eIF4A2, RACK1, RBP4, and PCBP1; from Sigma (Saint Louis, MO, USA), HA, eEF1A1, CES1, eIF3A, TUBB, and RNF187; from Biospes (Chongqing, China), ACTG1, C4a, C8, eIF4G, and TSPAN7; from St. John’s Laboratory (London, United Kingdom), BTBD6 and golginB1; from Abcam, Inc. (Cambridge, United Kingdom), eIF4E; from Life Technologies, Inc. (Carlsbad, CA), anti-rabbit Alexa Fluor 488, anti-rabbit Alexa Fluor 594, and Prolong Gold Antifade Mountant with DAPI (4′,6-diamidino-2-phenylindole); and from English and Scientific Consulting Kft. (Szirák, Hungary), monoclonal anti-dsRNA J2. Antibodies against ORF4 and X were generated as previously described (13, 33). Thymidine and propidium iodide (PI) were from Himedia Laboratories (Mumbai, India). Flag M2-agarose and HA-agarose were from Sigma (Saint Louis, MO, USA), and Myc-agarose was from Santa Cruz Biotechnology (Dallas, TX). Glutathione agarose, AHA (l-azidohomoalanine) (C10102), and a biotin protein analysis detection kit (C33372) were from Thermo Scientific (Waltham, MA). Control siRNAs (SC-37007) or target-specific siRNAs (pools of three target-specific siRNAs) against eIF2AK4 (SC-45644), eIF4A2 (SC-40556), RACK1 (SC-36354), eIF3A (SC-40547), and ACTG1 (SC-105037) were from Santa Cruz Biotechnology (Dallas, TX).

### Plasmids, viral RNA, cell culture, transfection, electroporation, infection, and cell cycle inhibition.

pGBKT7 vector expressing the 10·g-1 HEV proteins has been described previously (13, 33). G-3 HEV ORF1 domains, ORF3, and ORF2 were PCR amplified from pSK HEV p6 (GenBank accession no. JQ679013.1) plasmid and cloned into the pGBKT7 vector, as described in Text S1 in the supplemental material. pGEX4T-1 ORF4, pUNO PCP-Flag, pUNO Y-HA, and cDNAs encoding the different human genes were cloned as described in Text S1. shRNA-encoding plasmid against eEF1A1 has been reported previously (13). HEV genomic RNA was *in vitro* synthesized, as described previously (59); size and integrity were monitored by formaldehyde agarose gel electrophoresis. Huh7 human hepatoma cells have been described previously (60). The cell line was originally obtained from the laboratory of C. M. Rice (61). ORF4-Huh7 cells have been reported previously (13). Cells were maintained in Dulbecco’s modified Eagle medium (DMEM) containing 10% fetal calf serum (FCS) and 50 IU/ml penicillin and streptomycin in 5% CO_2_. Plasmids were transfected using Lipofectamine 2000, following the protocol of the manufacturer (Life Technologies, Inc., Carlsbad, CA). For siRNA transfection, Huh7 cells were seeded at ~80% confluence in a 12-well plate 16 to 18 h postseeding, A 10-μl volume of 10 μM siRNA was transfected using Lipofectamine 3000 (Life Technologies, Inc., Carlsbad, CA), followed by a second round of siRNA transfection after 24 h. At 24 h after the second transfection, cells were transferred into a 60-mm-diameter plate. Whole-cell extracts were prepared 48 h later, aliquots were used in Western blotting (to check silencing efficiency), and the remaining extract was stored at −80°C for future use in pulldown and immunoprecipitation assays. In experiments involving detection of viral proteins or HEV genomic RNA in siRNA-transfected cells, Huh7 cells expressing the viral RNA or plasmids were reseeded at ~70% confluence and transfected with the siRNAs followed by harvesting of cells after 2 or 4 days, respectively. For virus infection studies, a g-1 HEV clinical isolate was used as described previously (34). Briefly, ORF4-Huh7 cells were infected with a viral suspension containing 8 × 10^6^ genome copies. After 72 h, cells were transfected twice with siRNAs/shRNA as described above. Cell cycle inhibition was carried out by a double thymidine block, as described previously (35). Details are provided in Text S1.

10.1128/mSystems.00135-17.1 TEXT S1  Supplemental methods. Download TEXT S1, PDF file, 0.1 MB. Copyright © 2018 Subramani et al. 2018 Subramani et al. This content is distributed under the terms of the Creative Commons Attribution 4.0 International license.

### Screening of yeast two-hybrid libraries.

Commercially available Mate & Plate cDNA libraries of human liver and fetal brain were screened against the viral proteins expressed in the Y2H gold strain, following the instructions of the manufacturer (Clontech, Mountain View, CA, USA). Details are provided in Text S1.

### Bioinformatics studies.

The experimentally identified virus-host PPI data set was visualized using cytoscape (version 3.1.0) (29). All analyses were performed using the experimentally verified data of human interactome, sourced from the HPRD (human protein reference database) (30). The Network Analyzer plugin in cytoscape was used to compute the topological parameters and centrality measures of the PPIN. Gene ontology “GO” analysis was performed using the BiNGO (biological networks gene ontology) (31) app in cytoscape. Benjamini-Hochberg false-discovery-rate correction was used; *P* values of <0.05 were considered significant. HEV-human protein-protein interaction analysis was also performed using the STRING database (32), with the following parameters: data source, experimental and curated databases; confidence level, highest (0.9); maximum number of interaction partners, 50 (H_50_). For depicting the function of host translation factors that interact with various HEV proteins, a translation factor pathway (identifier [ID] WP107) was sourced from WikiPathways and imported into cytoscape. Nodes targeted by HEV proteins were highlighted. Primary interaction partners and secondary interaction partners are denoted by red and blue, respectively. Yellow denotes the target of eIF2AK4. A Web-based tool, Venny 2.1.0 (http://bioinfogp.cnb.csic.es/tools/venny/index.html), was used to generate the Venn diagram for comparing the Y2H data set (this study) with the profiles of differentially expressed proteins identified in HEV-infected human, pigs, and A549 cells (24–26).

### Protein purification and pulldown assay.

Soluble protein fractions of *E. coli* BL-21pLyS expressing GST-X were bound to glutathione-agarose beads in binding buffer (50 mM Tris-Cl [pH 8.0], 500 mM NaCl, 1 mM phenylmethylsulfonyl fluoride [PMSF], 1 mM dithiothreitol [DTT]). After three washes with wash buffer (binding buffer–0.1% Triton X-100) were performed, GST-X-bound beads were resuspended in thrombin cleavage buffer (20 mM Tris [pH 8.0], 150 mM NaCl, 2.5 mM CaCl_2_) and incubated with thrombin at 4°C for 16 h. The reaction was stopped by adding 1 mM PMSF (phenylmethyl sulfonyl fluoride). The supernatant was passed through a 10-kDa Centricon filter unit, and purified X was collected as the eluate. GST-ORF4-bound beads were washed three times in wash buffer. Soluble protein fractions of *E. coli* BL21(DE3)-C41 expressing GST-ORF4 were bound to glutathione-agarose beads in binding buffer (50 mM Tris-Cl [pH 7.4], 0.25% sucrose, 150 mM NaCl, 0.5% NP-40, 1 mM PMSF). After three washes performed with wash buffer (50 mM Tris-Cl [pH 7.4], 150 mM NaCl, 1% Triton X-100), GST-ORF4-bound beads were used directly for the pulldown assay or GST-ORF4 was eluted from the beads with elution buffer (50 mM Tris [pH 7.4], 150 mM NaCl, 20 mM glutathione). Histidine-tagged methyltransferase and ORF3 were purified from *E. coli* Rosetta pLysS and BL21(DE3) cells, respectively, as described previously (33). C-terminal Flag-tagged PCP, V, helicase, ORF2, and RdRp proteins were expressed in Huh7 cells, followed by preparation of whole-cell extract and incubation with Flag M2 agarose beads. Flag-tagged proteins were selectively eluted by the use of an excess of Flag peptide, following the guidelines of the manufacturer (Sigma, Saint Louis, MO, USA). C-terminal HA-tagged Y was expressed in Huh7 cells, followed by preparation of whole-cell extract and incubation with HA-agarose beads. HA-tagged proteins were selectively eluted by an excess of HA peptide, following the guidelines of the manufacturer (Sigma, Saint Louis, MO, USA). N-terminal myc-tagged RdRp protein was expressed in Huh7 cells, followed by preparation of whole-cell extract and incubation with myc agarose beads. Myc-RdRp protein was eluted by incubation with 100 mM glycine (pH 2.5), purity was checked by silver staining using a commercially available kit (Thermo Scientific, Waltham, MA), and specificity was checked by Western blotting using Myc (9E10) antibody. For pulldown assays, RdRp-myc bound to the beads was used directly (without elution).

Approximately 1 μg of *E. coli* purified viral proteins, 4 μg of Flag-agarose or HA-agarose affinity purified proteins, and 1 mg of whole-cell extract of Huh7 cells (wild type or siRNA/shRNA transfected) were incubated with glutathione agarose or myc-agarose-bound GST-ORF4 and RdRp-myc proteins, respectively, for 2 h, on a flip-flop rocker at 4°C. Beads having only GST or lacking RdRp-myc were processed in parallel as a mock treatment. Beads were washed three times in IP buffer (20 mM Tris [pH 7.4], 150 mM NaCl, 1 mM EDTA [pH 8.0], 1 mM EGTA [pH 8.0], 1% Triton X-100, 2.5 mM sodium pyrophosphate, 1 mM β-glycerol phosphate, 1 mM sodium orthovanadate), followed by addition of 3× Laemmli buffer (187.5 mM Tris [pH 6.8], 6% SDS, 30% glycerol, 150 mM DTT, 0.03% bromophenol blue) and incubation at 95°C for 5 min. Flag-tagged RdRp, PCP, helicase, and V were detected using Flag antibody. His-tagged methyltransferase and ORF3 proteins were detected using His antibody. Y-HA was detected using HA antibody. ORF4, X, eIF4A2, eIF4E, eIF4G, eIF3A, eEF1A1, RACK1, eIF2AK4, TUBB, and ACTG1 proteins were detected using corresponding antibodies.

### Total RNA isolation, quantitative real-time PCR, RdRp assay, immunoprecipitation, immunofluorescence assay, silver staining, Western blotting, cell viability assay, and luciferase assay.

Total RNA was isolated using TRI reagent (MRC Inc., Cincinnati, OH). Reverse transcription was done using SuperScript III (Life Technologies, Inc., Carlsbad, CA), following the manufacturer’s instructions, followed by quantitative real-time PCR, as described previously (13). To measure HEV sense and GAPDH RNA levels, cDNA was synthesized using random hexamers. To measure HEV antisense RNA levels, an HEV antisense RT primer (5′-CGTGTCATGGTGGCGAATAAGCAGACCACATATGTGGTCGAT-3′) was used for cDNA synthesis. The following primers were used for quantitative real-time PCR: for HEV sense FP, 5′-CGGCCCAGTCTATGTCTCTG-3′; for HEV sense RP, 5′-TAGTTCCTGCCTCCAAAAAG-3′; for HEV antisense FP, 5′-GTGTCATGGTGGCGAATAAG-3′; for HEV antisense RP, 5′-AACGGTGGACCACATTAGGA-3′; for hGAPDH FP, 5′-GAGTCAACGGATTTGGTCGT-3′; for hGAPDH RP, 5′-TTGATTTTGGAGGGATCTCG-3′. HEV sense and antisense RNA levels were normalized to that of GAPDH (glyceraldehyde-3-phosphate dehydrogenase) and are presented as means ± standard errors of the means (SEM) of values from three experiments, analyzed using GraphPad Prism software and the Student *t* test. *P* values of <0.05 were considered significant. The cell viability assay was done using a CellTiter 96 non-radioactive cell proliferation assay {MTT [3-(4,5-dimethyl-2-thiazolyl)-2,5-diphenyl-2H-tetrazolium bromide]} kit (Promega, USA), as described previously (34). *Renilla* luciferase activity in culture medium was measured using a *Renilla* luciferase assay kit (Promega, USA). RdRp assays, immunoprecipitation, immunofluorescence assays, silver staining, and Western blotting were done as described previously (13). Details are provided in Text S1.

### *In vitro* coupled transcription-translation (TNT) assay and immunoprecipitation.

A TNT T7 kit was used for *in vitro* synthesis of ORF1 protein, following the manufacturer’s instructions. Briefly, 1 µg of pGBKT7 (mock treatment) and pGBKT7 ORF1 plasmid DNA was added to the reaction tube containing TNT Quick Master Mix and 1 mM methionine. The reaction mixture was incubated at 30°C for 90 min, and 3-µl aliquots were used to detect the ORF1 protein by Western blotting using anti-Flag antibody. For IP using Flag agarose beads, 6 µl lysate was mixed with 1 ml Huh7 cell lysate (2 million cells lysed in 1 ml IP buffer), 1 µl purified GST-ORF4 protein (200 ng/µl), and 80 µl Flag agarose beads (50% solution) and incubated on a flip-flop rocker for 16 h at 4°C. Beads were washed three times in IP buffer, followed by incubation with 200 μl Flag peptide (0.2 mg/ml in phosphate-buffered saline [PBS]) on a flip-flop rocker for 15 min at 4°C. Supernatant containing the eluted proteins was collected, and aliquots were subjected to Western blotting with different antibodies, as indicated.

### Global translation measurement by l-azidohomoalanine labeling.

Labeling of nascent proteins was done using nonradioactive Click-IT l-azidohomoalanine (AHA). Huh7 cells were transfected with different siRNAs and shRNA and incubated for 72 h. Cells were washed in PBS and incubated in methionine-free media for 1 h. AHA (50 µM) was added into each plate and incubated for 2 h followed by harvesting and three washes in PBS. The pellet was lysed in lysis buffer (1% SDS, 50 mM Tris-HCl [pH 8.0], 1× protease inhibitor cocktail) on ice for 1 h and sonicated for 5 s, and an equal amount of protein in the supernatant was used for Click-IT reaction, following the instructions of the manufacturer (Click-IT biotin protein analysis detection kit). The final protein pellet was lysed in 50 µl of Laemmli buffer and subjected to Western blotting using streptavidin-horseradish peroxidase (HRP).
