# Supplementary material for: Host-Virus Protein Interaction Network Reveals the Involvement of Multiple Host Processes in the Life Cycle of Hepatitis E Virus
Source: mSystems. 2018 Jan 23;3(1):e00135-17. doi: 10.1128/mSystems.00135-17 (PMC5781259; doi:10.1128/mSystems.00135-17)
Supplement: TABLE S1 [file sys001182163st1.pdf]

TABLE S1. Estimation of mating efficiency between bait and prey strains.

Viral bait	Cell density of bait culture	Cell density of prey culture	No. of cfu/ml on T^-	No. of cfu/ml on L^-	No. of cfu/ml on LT^-	% Mating efficiency	No. of diploid clones screened (million)
**a. Estimation of mating efficiency between Y2H gold (g-1 HEV proteins) and Y187 (human fetal brain cDNA library)**							
**Met**	$1.4*10^8$	$6.1*10^7$	$8.4*10^7$	$1.3*10^6$	$1.8*10^4$	1.3	0.20
**Y**	$1.2*10^8$	$6*10^7$	$8.4*10^7$	$2*10^6$	$8.4*10^4$	1.7	0.39
**PCP**	$1.1*10^8$	$3.8*10^7$	$2.6*10^7$	$2*10^6$	$1.7*10^5$	8.85	2.03
**V**	$1.5*10^8$	$3.2*10^7$	$5.8*10^7$	$1.3*10^6$	$1*10^5$	7.2	1.1
**X**	$1*10^8$	$4*10^7$	$4*10^8$	$8*10^7$	$5.3*10^5$	0.6	6
**Helicase**	$1.1*10^8$	$3*10^7$	$9.5*10^7$	$1.5*10^6$	$4.5*10^4$	2.9	0.51
**RdRp**	$1.1*10^8$	$3.5*10^7$	$9.4*10^7$	$1.8*10^6$	$4.3*10^4$	2.3	0.5
**ORF2**	$1.3*10^8$	$3*10^7$	$7.2*10^7$	$2.5*10^6$	$3.8*10^4$	2.7	2.4
**ORF3**	$1*10^8$	$2*10^7$	$1.8*10^8$	$1.6*10^6$	$8.5*10^4$	5.3	.96
**ORF4**	$1.1*10^8$	$3.2*10^7$	$9.1*10^7$	$1.65*10^7$	$2.1*10^4$	1.2	0.24
**b. Estimation of mating efficiency between Y2H gold (g-1 HEV proteins) and Y187 (human liver cDNA library)**							
**Met**	$1.1*10^8$	$1*10^8$	$1.4*10^8$	$2.7*10^6$	$1.3*10^6$	5.03	1.5
**Y**	$1*10^8$	$9*10^7$	$4.6*10^7$	$7*10^6$	$2.7*10^5$	3.8	3.1
**PCP**	$1.1*10^8$	$3.4*10^7$	$4.7*10^7$	$2.8*10^6$	$4.6*10^5$	1.6	5.29
**V**	$1*10^8$	$1*10^8$	$3.1*10^7$	$1.5*10^6$	$2.3*10^5$	15.3	2.6
**X**	$1.1*10^8$	$7.5*10^7$	$4.3*10^7$	$3.1*10^6$	$3*10^5$	9.8	3.5
**Hel**	$1.1*10^8$	$1*10^8$	$1.3*10^8$	$3.2*10^6$	$8.9*10^5$	2.7	1.02
**RdRp**	$1*10^8$	$6.5*10^7$	$3.7*10^7$	$3.7*10^6$	$1.4*10^5$	4	1.7
**ORF2**	$1.4*10^8$	$4*10^7$	$8.5*10^7$	$7.2*10^6$	$2*10^5$	1.52	4.3
**ORF4**	$1*10^8$	$3*10^7$	$9.64*10^7$	$1*10^6$	$2.8*10^4$	2.5	0.32

L: Leucine, T: Tryptophan, "-": Deficiency in the medium, "+": Supplemented in the medium, cfu: colony forming unit.
