# Supplementary figures and images for: Host-Virus Protein Interaction Network Reveals the Involvement of Multiple Host Processes in the Life Cycle of Hepatitis E Virus
Source: mSystems. 2018 Jan 23;3(1):e00135-17. doi: 10.1128/mSystems.00135-17 (PMC5781259; doi:10.1128/mSystems.00135-17)

FIG S1

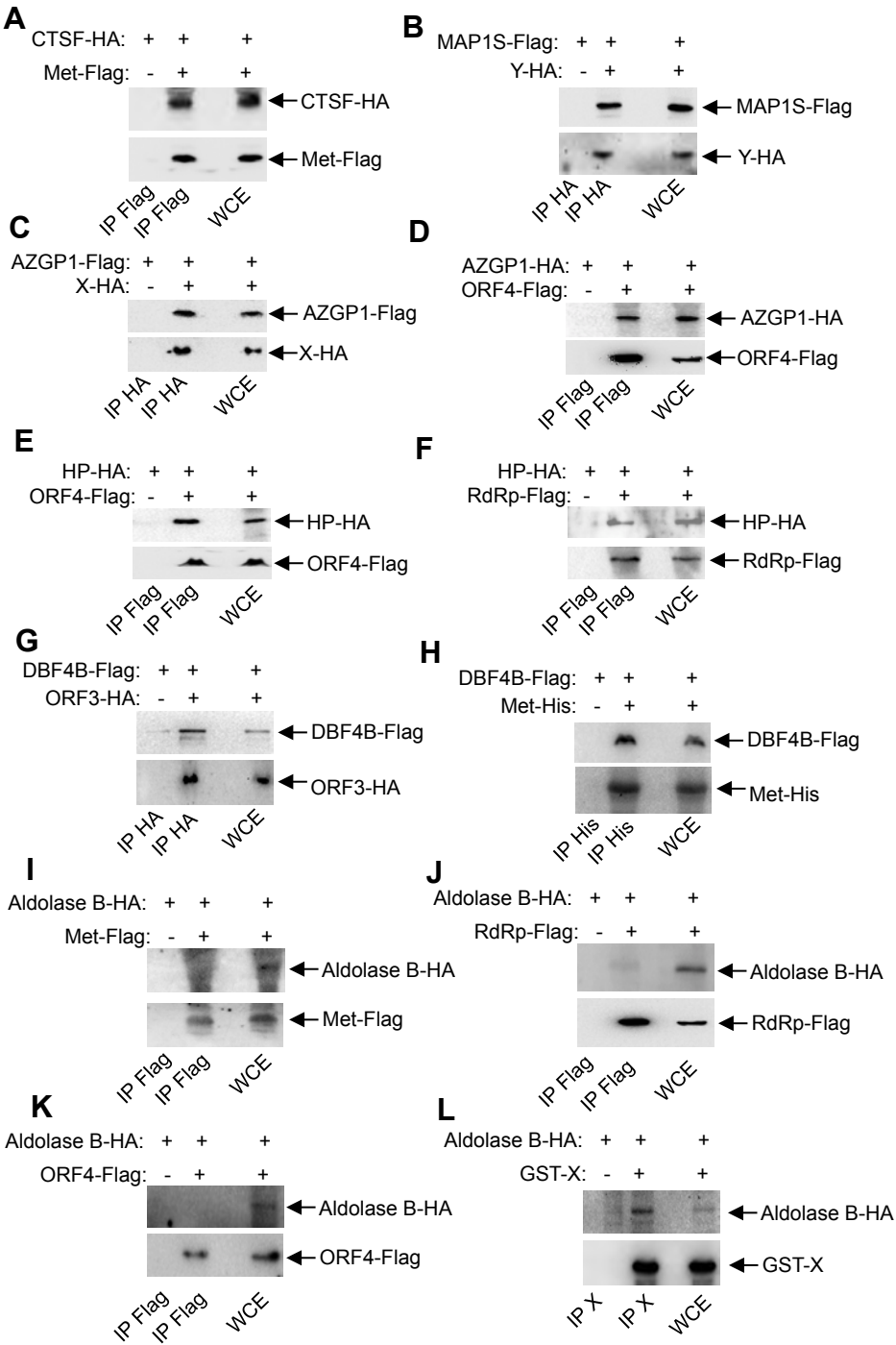

Supplement: FIG S1 [file sys001182163sf1.pdf]

FIG S2

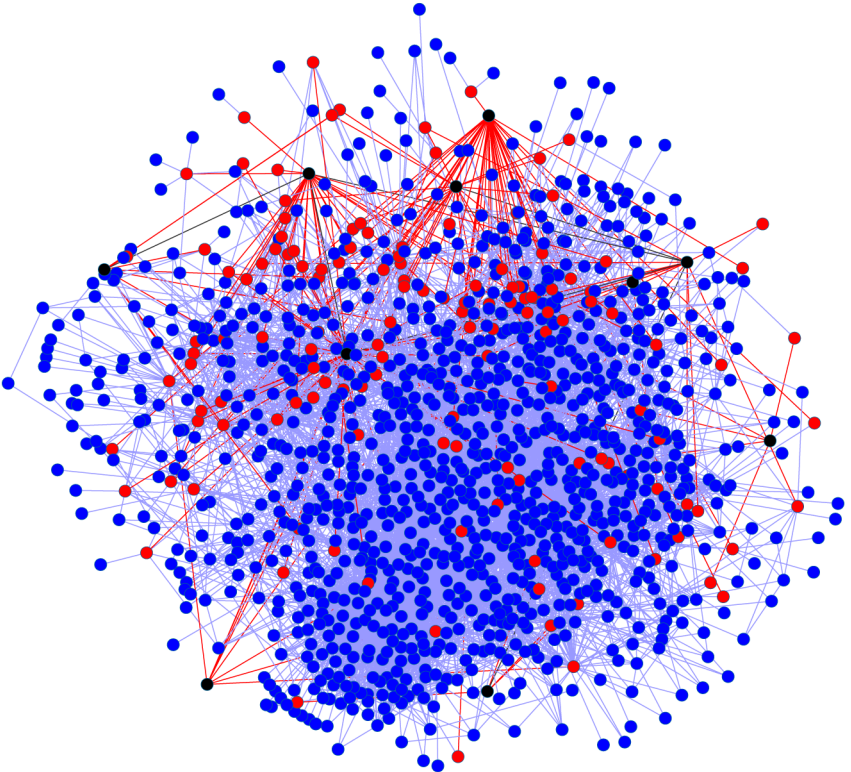

Supplement: FIG S2 [file sys001182163sf2.pdf]

FIG S3

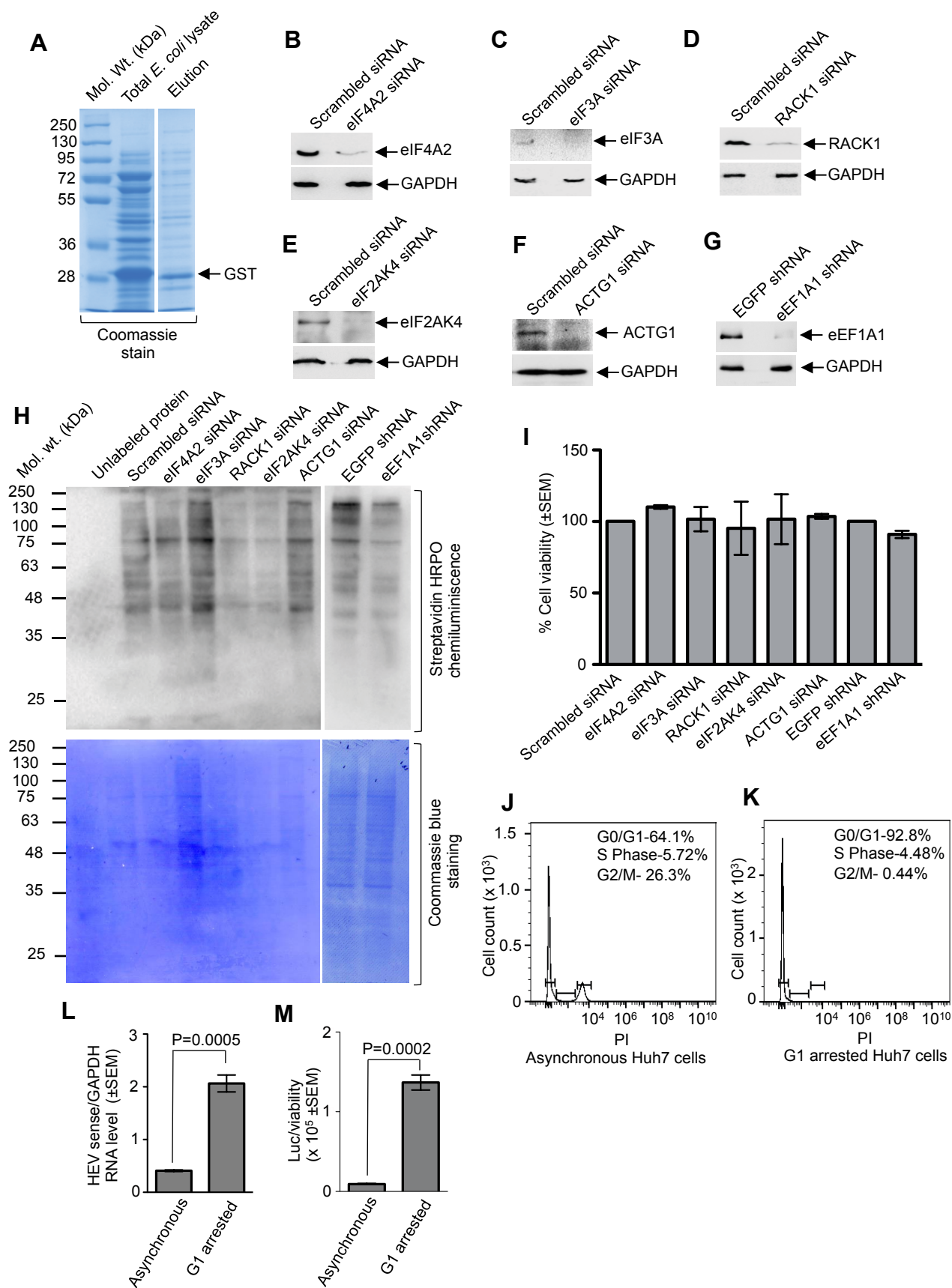

Supplement: FIG S3 [file sys001182163sf3.pdf]

FIG S4

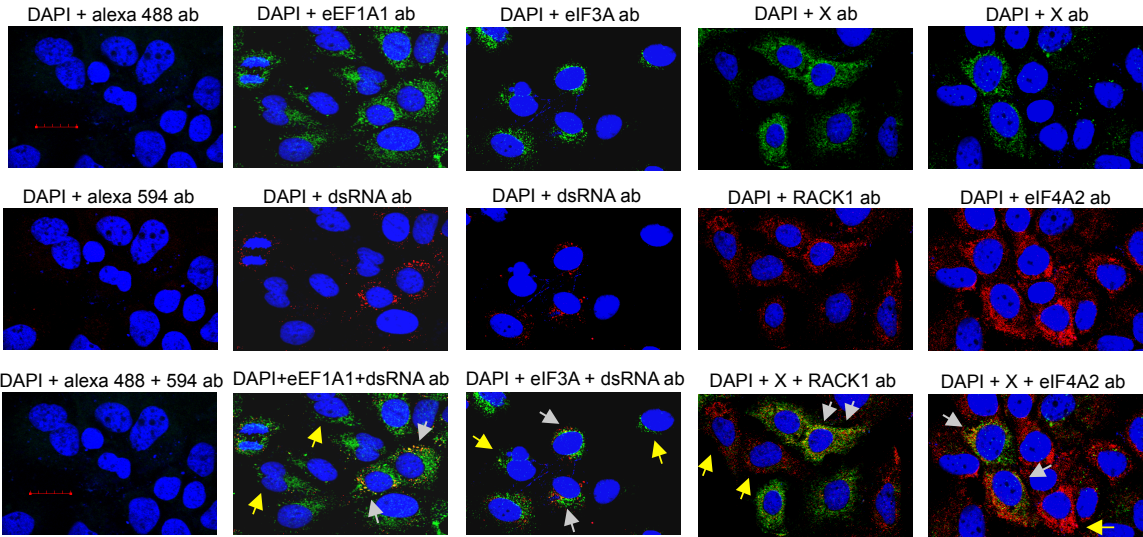

Supplement: FIG S4 [file sys001182163sf4.pdf]
