# Supplementary material for: Host-Virus Protein Interaction Network Reveals the Involvement of Multiple Host Processes in the Life Cycle of Hepatitis E Virus
Source: mSystems. 2018 Jan 23;3(1):e00135-17. doi: 10.1128/mSystems.00135-17 (PMC5781259; doi:10.1128/mSystems.00135-17)
Supplement: TEXT S1 [file sys001182163s1.pdf]

Supplementary methods

Cloning

Following clones were generated, as described below. All clones were confirmed by restriction mapping and sequencing of the DNA.

pGBKT7 g-3 Methyltransferase	g-3 Methyltransferase sequence (nt 126-1091 from 5' end) was PCR amplified with forward primer 5'-TCGCCGGAATTCGAGGCCCACCAGTTCATTAAG -3' and reverse primer 5'- AGACTAGTCGACGATCCACGCACGAAGTATGG -3' using pSK HEV p6 as template. The PCR product was digested with EcoRI and SalI and ligated with EcoRI and SalI digested pGBKT7 vector.
pGBKT7 g-3 Y	g-3 Y sequence (nt 672-1352) was PCR amplified with forward primer 5'- ATGCGAATTCCGCGCCGTTGTAACCT-3' and reverse primer 5'-ATGCGTCGACTTAGAATCCTGCAGATAGCC-3' using pSK HEV p6 as template. The PCR product was digested with EcoRI and SalI and ligated with EcoRI and SalI digested pGBKT7 vector.
pGBKT7 g-3 PCP	g-3 PCP sequence (nt 1320-1802) was PCR amplified with forward primer 5'- ATGCGAATTCGCACAGTGCCGACGGTGG-3' and reverse primer 5'- ATGCGGATCCTTACAGGACATACTGCTCTGGGCCA -3' using pSK HEV p6 as template. The PCR product was digested with EcoRI and BamHI and ligated with EcoRI and BamHI digested pGBKT7 vector.

pGBKT7 g-3 V	g-3 V domain (Accession number: AB291955.1, nt 2124-2360) region "CCCGGGGACGTCTGGTTTTTCCAGTGACTTTTCCCCTCCTGAGGTGGCCACCCTTGCACCGGCCGCTGCCCCTGGGCTGCCCCACTCTACCCCACCTGTCAGTGATATTTGGGTGTTACCACCGCCCTCAGAAGAGTCTCAGGTTGATGCGGCTCCTGCACTTCCTGCCCCCGAGCCCGTTGGGCTGCCCAGCCCTGTTAAGCTTACTCCCCCCGTGCGTAAGCCACCGGCATCGCCGCCTTCTGTCGAC" was synthesized along with SmaI and SalI sites and cloned into pGBKT7 vector between SmaI and SalI sites.
pGBKT7 g-3 X	g-3 X sequence (nt 2610-2948) was PCR amplified with forward primer 5'- ACGCTCATATGGTGTATGCAGGGTCATTGTTTG-3' and reverse primer 5'- ATGCGGATCCACCCAGGCATCAAAACTGAG-3' using pSK HEV p6 as template. The PCR product was digested with NdeI and BamHI and ligated with NdeI and BamHI digested pGBKT7 vector.
pGBKT7 g-3 Helicase	g-3 Helicase sequence (nt 3120-3854) was PCR amplified with forward primer 5'- ACGCTGAATTCGGCTGCACTATCAGTCCTG-3' and reverse primer 5'- ATGCGGATCCAGAAAAAATTATTGACAATCACATCCG -3' using pSK HEV p6 as template. The PCR product was digested with EcoRI and BamHI and ligated with EcoRI and BamHI digested pGBKT7 vector.
pGBKT7 g-3 RdRp	g-3 RdRp sequence (nt 3861-5321) was PCR amplified with forward primer 5'- ATGCGAATTCGGCGGAGAGGTCGGCC-3' and reverse

	primer 5'- ATGCGTCGACTTATTCTACCCGCTGTATGATGGAAT-3' using pSK HEV p6 as template. The PCR product was digested with EcoRI and SalI and ligated with EcoRI and SalI digested pGBKT7 vector.
pGBKT7 g-3 ORF2	g-3 ORF2 sequence (nt 5692-7182) was PCR amplified with forward primer 5'- ATGCCATATGGCTGTATCACCAGCCCC-3' and reverse primer 5'- ATGCGTCGACTTAAGCAAGGGCCGAGTGTG-3' using pSK HEV p6 as template. The PCR product was digested with NdeI and SalI and ligated with NdeI and SalI digested pGBKT7 vector.
pGBKT7 g-3 ORF3	pUNO ORF3 geno3 (nt 5321-5689) DNA was digested with AgeI and NheI and end filled. ORF3 band was gel extracted and ligated with pGBKT7 vector that was digested with NcoI and end filled.
pUNO g-1 ORF1 Flag	pUNO-hIPSI (Invivogen) was digested with AgeI and NheI and vector backbone (2908 bp) was gel extracted. ORF1 genotype1 sequence (nt 125-5107) was PCR amplified with forward primer 5' GACACCGGTCATCATGGAGGCCCATCAGTTTATCAAGGC-3' and reverse primer 5'- GACGCTAGCTCACTTATCGTCATCGTCCTTGTAGTCTTCCACCCGACACAGAATTG-3' using pSK HEV2 as template and digested with AgeI and NheI. 1894 and 3089 base pair fragments were gel extracted and ligated with pUNO vector.
pGBKT7 g-1	From methyltransferase to X spanning region (nt 125-2851) was PCR

ORF1 Flag	amplified with forward primer 5'- TGCTCAGAATTCGTTAGGCCTTTTCTCTCTC-3' and reverse primer 5'- AGACTGCTCGAGTTATGCTGTCCGCGCAAC-3' using pSK HEV2 as template. The PCR product was digested with EcoRI and XhoI and ligated with EcoRI and SalI digested pGBKT7 vector. This clone was named as pGBKT7 Met-X.<br><br>From X to RdRp spanning region (nt 2378-5107) was PCR amplified with forward primer 5'- TGCTCAGAATTCCCGGATGGCTCTAAGGTG-3' and reverse primer 5'- GACGCTAGCTCACTTATCGTCATCGTCCTTGTAGTCTTCCACCCGACACAGAATTG-3' using PSK HEV2 as template. The PCR product was digested with MluI and 2669 bp fragment was gel extracted. pGBKT7 Met-X was digested with NotI, blunted, digested with MluI and a 9615 bp fragment was gel extracted. 2669 base pair insert was ligated with 9615 base pair vector.
pGEX4T1 ORF4	ORF4 sequence (nt 2838-3308) was PCR amplified with forward primer 5'- GATCAGAATTCTTGCGCGGACAGCAAATCT-3' and reverse primer 5'- AGATCGAATTCGCTCACATACATCCGCAGG-3' using pSK HEV2 as template. The PCR product was digested with EcoRI and ligated with EcoRI digested pGEX4T1 vector.
pUNO g-1 PCP-Flag	pUNO-hIPSI (Invivogen) was digested with AgeI and NheI and vector backbone (2908 bp) was gel extracted. g-1 PCP sequence (nt 1322-

	1801) was PCR amplified with forward primer 5'-GACACCGGTCATCATGCAGTGTAGGCGCTGGCTCTC-3' and reverse primer 5'-GACGCTAGCTCACTTATCGTCATCGTCCTTGTAGTCGAGATTGTGGCGCTCTGG -3' using pSK HEV2 as template. The PCR product was digested with AgeI and NheI and ligated with vector.
pUNO g-1 Y-HA	pUNO-hIPSI (Invivogen) was digested with AgeI and NheI and vector backbone (2908 bp) was gel extracted. g-1 Y sequence (nt 671-1351) was PCR amplified with forward primer 5'-GACACCGGTCATCATGCGCGTTGTGGTGACGTATGAGG -3' and reverse primer 5'-GACGCTAGCTCAAGCGTAATCTGGAACATCGTATGGGTAAAAGCCGGCCGAGAGCCAGCGCCTACAC -3' using pSK HEV2 as template. The PCR product was digested with AgeI and NheI and ligated with vector.
pCDNA5 ALDOB-HA	ALDOB sequence was PCR amplified with forward primer 5'-CTATTCGATGATGAAGATACCCCACCAAACCCA-3' (FP AD) and reverse primer 5'-ATGCGTTTAAACGCGGCCGCGTGAACTTGCGGGGTTTTTCAGTATCTACGAT -3' (RP RecAB) using pGADT7 RecAB ALDOB as template. The PCR product was digested with NotI and ligated with EcoRV and NotI digested pCDNA5 vector.

pCDNA5 CTSF-HA	CTSF sequence was PCR amplified with FP AD and RP RecAB primers using pGADT7 RecAB CTSF as template. The PCR product was digested with NotI and ligated with EcoRV and NotI digested pCDNA5 vector.
pCDNA5 HP-HA	HP sequence was PCR amplified with FP AD and RP RecAB primers using pGADT7 RecAB HP as template. The PCR product was digested with NotI and ligated with EcoRV and NotI digested pCDNA5 vector.
pCDNA5 AZGP1-HA	AZGP1 sequence was PCR amplified with FP AD and RP RecAB primers using pGADT7 RecAB AZGP1 as template. The PCR product was digested with NotI and ligated with EcoRV and NotI digested pCDNA5 vector.
pUNO AZGP1-Flag	AZGP1 sequence was PCR amplified with forward primer 5’-ATCGGTCGACATGGACTACAAGGACGACGATGACAAGGGTTGTGAGATCGAGAAT-3’ and reverse primer 5’-ATGCGCTAGCGAAGTGAACTTGCGGGGT-3’ (RP RecAB1) using pGADT7 RecAB AZGP1 as template. The PCR product was digested with SalI and NheI and ligated with SalI and NheI digested pUNO vector.
pUNO MAP1S-Flag	MAP1S sequence was PCR amplified with forward primer 5’-ATCGGTCGACATGGACTACAAGGACGACGATGACAAGGATCCCGTGCCCCTGG -3’ and RP RecAB1 using pGADT7 RecAB MAP1S as template. The PCR product was digested with SalI and NheI and ligated

	with SalI and NheI digested pUNO vector.

Yeast two hybrid (Y2H) library screening

A GAL4 based Y2H assay was used for screening commercially available mate and plate human fetal brain and liver yeast two hybrid cDNA libraries against all g-1 HEV proteins. Libraries were pretransformed into *S. cerevisiae* Y187 (mating type α) host. Bait clones were expressed in Y2H gold (mating type a). Y2H gold contains four reporter genes under three different GAL4 responsive promoters for monitoring protein-protein interaction. The four reporter genes are (a) *AUR1-C* codes for inositol phosphoryl ceramide synthase, confers resistance to Aureobasidin A (b) *HIS3* expression permits the cell to grow on histidine auxotrophic plate (c) *ADE2* expression permits the cell to grow on adenine auxotrophic plate (d) *MEL1* codes for α-galactosidase that cleaves chromogenic substrate X- α -Gal and displays blue color.

a) Self-activation test of bait proteins

To test whether the bait proteins activate the reporters in the absence of prey, Y2H gold strain were transformed with the activation domain (AD) vector pGADT7 and respective bait protein encoding plasmids. Eight random colonies from each transformation were replica plated onto plates containing the following medium to monitor growth and color of the colonies: $LTHA^-$ (Leu, Trp, His, Adenine deficient standard dropout medium), LTH^- with Aureobasidin A [(A^{r+}) 200ng/ml], LT^- with X-α-Gal (400µg X-α-Gal in DMF/ 90mm plate) and LTH^- with 5, 10 and 20mM of 3 amino-1, 2, 4-triazole (3-AT). Note that 3-AT is a competitive inhibitor of the enzyme imidazoleglycerol-phosphate dehydratase

(encoded by the *HIS3* reporter gene), which catalyzes the sixth step in histidine biosynthesis pathway. A high affinity interaction will produce more imidazoleglycerol-phosphate dehydratase, which in turn requires higher quantity of 3-AT to be inhibited. Hence, ability of the cotransformants to grow on increasing amount of 3-AT indicates the strength of interaction of the test proteins.

Except RdRp, other HEV proteins displayed poor growth on LTH$^-$ medium (denoted by "+"). No other reporters were positive (Data is not shown). RdRp activated all reporters to some extent (Data is not shown). Based on the above observation, RdRp interacting partners were initially screened on LTHA$^-$ + 10mM 3-AT plates; primary screening of all the other baits were performed on LTHA$^-$ plates.

b) Library screening

Y2H Gold culture expressing the respective bait proteins were grown to a cell density of >1x10^8 cells/ml, as recommended by the manufacturer (numbers were estimated by cell counting using haemocytometer, individual values mentioned in Table S1). Density of Y187 cells containing the library clones were counted (as instructed in the brochure) to ensure that they have the minimum recommended density, which is >2x10^7 cells/ml (individual values mentioned in Table S1). Bait and prey containing hosts were allowed to mate in rich medium (2X YPDA+ 50µg/ml Kanamycin) for 20 hours. Diploids were plated on indicated mediums (based on the self-activation test result). Mating efficiency was calculated following the instruction of the manufacturer. Five days post-incubation of the plates at 30^{0}C in a humidified incubator, colonies were transferred to LT$^-$ plates followed by replica plating onto LT$^-$, LTH$^-$, LTHA$^-$, LTH$^-$A^{r+}, LT$^-$+ X-α gal, LTH$^-$+ 5mM 3-AT, LTH$^-$+ 10mM 3-AT and LTH$^-$+ 20mM 3-AT plates. Colonies that activated all

reporters were streaked three times on LT^-+ X-α gal plates to segregate and isolate individual plasmids. Final streaking was done on $LTHA^-$+ X-α gal +A^{r+} (For RdRp, 10mM 3-AT was supplemented in the medium) to eliminate false positives or non-performing clones (Table 1). The prey cDNA containing pGADT7-Rec plasmids were isolated using zymolase and alkaline lysis method, followed by transformation into *E.coli* TOP10 and amplification of DNA. Restriction mapping was done using EcoRI, SacI and HindIII, PstI combinations to eliminate identical plasmids. To further eliminate false-positives, retransformation assay was performed. Y2H Gold cells were cotransformed with bait and prey plasmids along with the appropriate control plasmids and replica plated onto LT^-, LTH^-, $LTHA^-$, LTH^-A^{r+}, LT^-+ X-α gal, LTH^-+ 5mM 3-AT, LTH^-+ 10mM 3-AT and LTH^- + 20mM 3-AT (Table S2A) medium. Prey plasmids that activated all four reporters only in the presence of the bait protein were sequenced by dideoxy chain termination method, using an automated sequencer.

c) Sequence analysis and selection of bonafide interaction partners

Insert sequences were analyzed using BLASTn (https://blast.ncbi.nlm.nih.gov/Blast.cgi?PAGE_TYPE=BlastSearch). Sequences were manually scrutinized and categorized into two groups: those containing protein coding sequence in frame with the BD and those containing 5’ UTR (with or without protein coding sequence), 3’ UTR, non-coding sequence and uncharacterized regions of chromosome. Only the clones containing coding sequence in frame with the BD were considered relevant (Table S3).

Protein purification

pGEX4T1-X was transformed into *E.coli* BL21 pLysS cells. 100 ml of culture was grown to A_{600} ~0.5 and induced with 1 mM of IPTG at 18^0C for 16 hours. Pellet was resuspended in 10 ml binding buffer [50mM Tris-Cl (pH 8.0), 500mM NaCl, 1mM PMSF, 1mM DTT] and treated with lysozyme (0.25 mg/ml) for 30 minutes. Sonication was done at 30 amplitude for 5 cycles (each cycle 5 sec on and 10 sec off). Sonicated lysate was centrifuged and soluble proteins were collected from the supernatant, followed by purification as described in methods.

pGEX4T1-ORF4 was transformed into *E.coli* BL21 C41 cells. 100 ml of culture was grown to A_{600} ~0.5 and induced with 0.1 mM of IPTG at 18^0C for 16 hours. Pellet was resuspended in 10 ml binding buffer [50mM Tris-Cl (pH 8.0), 0.25% sucrose, 150mM NaCl, 1mM PMSF, 0.5% NP-40] and treated with lysozyme 0.25 mg/ml for 30 minutes. Sonication was done at 30 amplitude for 5 cycles (each cycle 5 sec on and 10 sec off). Sonicated lysate was centrifuged and soluble proteins were collected from the supernatant, followed by purification as described in methods.

RdRp assay

3 µg pUNO RdRp-Flag was transfected into wild type, siRNA (eIF4A2, eIF3A, RACK1, eIF2AK4 and ACTG1) or eEF1A1shRNA transfected Huh7 cells (see Fig. 5C). 48 hours post-transfection, whole cell extract was prepared and RdRp was affinity purified using Flag M2-agarose beads, as described previously (13). Protein amount was equalized between the different samples by Bradford assay and visualized by western blotting of aliquots, using Flag antibody. Equal amount of protein was used for the assay, following previously described protocol (13). For RdRp assay to evaluate the effect of viral factors on the activity of the former, approximately 5 µg of affinity purified RdRp protein was

mixed with 5 μg of affinity purified ORF4, PCP, X or V domain proteins (in different combinations, as indicated in Fig 5D) and assay was carried out.

HEV specific 340 nucleotide ssRNA template, which was used as template in RdRp assays to monitor anti-sense strand synthesis was synthesized using mMessage mMachine T7 kit (Ambion, USA) as described previously (13, 36). The conditions for RdRp assay were similar to that described previously (13, 36). The RNA product was resolved on 2% formaldehyde agarose gel and northern blotting was carried out. The incorporation of digoxigenin (DIG) labeled UTPs in negative RNA strand was detected using CDP-star (Roche, USA).

Inhibition of Huh7 cell cycle at G0/G1 phase by double Thymidine block

Double Thymidine block protocol was followed to arrest the Huh7 cells at G0/G1 phase of the cell cycle, as reported previously (35). Briefly, Huh7 cells were treated with 2mM Thymidine for 19 hours, followed by three washes with PBS and incubated in fresh media (without Thymidine) for 9 hours. Cells were again treated with 2mM Thymidine for 16 hours, followed by harvesting in trypsin and washing once with PBS. The cells were fixed in 70% ethanol for 30 minutes at 4^0 C, followed by two washes with PBS. The cell pellet was resuspended in 200 μl of PBS, followed by treatment with 4 μl of Rnase A (10mg/ml) for 30 minutes at 37^0 C. 8 μl of 1mg/ml propidium Iodide (PI) solution was added to the suspension and cells were sorted by FACS (fluorescence activated cell sorting) in BD FACSCanto (BD Biosciences, USA).

Similar condition was followed to induce G0/G1 arrest in Huh7 cells expressing g-1 or g-3 HEV as described below. Huh7 cells were transfected with g-1 HEV RNA using lipofectamine 3000 or electroporated (Bio-Rad Gene Pulser Xcell; Bio-Rad, USA) with g-3 p6 HEV-Luc RNA in a 4 mm cuvette at 200 V, 950 μF, and infinite resistance (34). On 5th day of transfection/electroporation, double Thymidine block was induced as described above with the following modifcation. After 44 hours of Thymidine block, media was replaced with fresh media having 2mM thymidine for another 24 hours, followed by isolation of total RNA and QRT-PCR analysis of g-1 HEV sense and GAPDH RNA levels or measurement of *Renilla* luciferase activity and cell viability of p6 HEV-Luc expressing cells. Asynchronous cells were processed in parallel as controls.

Immunofluorescence assay (IFA)

6 μg of *in vitro* transcribed, capped g-1 HEV genomic RNA was transfected into Huh7 cells in 12 well plate at ~70% confluency using lipofectamine 3000. 24 hrs post transfection, culture medium was replaced with fresh medium. 48 hrs post transfection, cells were transferred into 60mm plate. On 5th day post-transfection, cells were seeded on coverslips in 12 well plate at ~50% confluency. 48 hrs post-seeding, cells were washed once in PBS followed by fixation in 1 ml 4% paraformaldehyde for 15 minutes at room temperature. After three washes in PBS, cells on coverslips were incubated with blocking solution (5% Normal Donkey Serum, 5% BSA in PBS, 0.3% Triton-X-100) for 60 minutes at room temperature. Coverslips were washed once in PBS and incubated with primary antibodies (in PBS, 0.3% Triton-X-100, 1% BSA) overnight at 4^{0}C. Primary antibody dilutions for eIF3A, eEF1A1, RACK1, eIF4A2 was 1:50 each. X antibody was diluted 1:100 and dsRNA J2 antibody was diluted 1:200. Next day, coverslips were

washed three times in PBS and incubated for 1 hour with 1:500 dilution of anti-rabbit alexa fluor-488, anti-rabbit alexa fluor-594 or anti-mouse alexa fluor-594 (in PBS, 0.3% Triton-X-100, 5% BSA). Coverslips were washed three times in PBS and mounted on glass slides using prolong gold antifade mountant with DAPI (4’, 6’-diamino 2-phenyl indole). DAPI, alexa fluor-488, alexa fluor-594 gives blue, green and red color, respectively. Antibodies for X, ORF2, eIF3A and eEF1A1 were raised in rabbit, shown in green color. Antibodies for RACK1, eIF4A2 and dsRNA J2 were raised in mouse, shown in red color. Anti-rabbit helicase antibody was revealed using goat anti-rabbit alexa fluor 594, visible in red color. Images were acquired using a 60X objective in a confocal microscope (Olympus 4.1 FV1000) and analyzed by fluoview software. Colocalization of green and red signal is visible as yellow color by superimposition of corresponding images, as indicated in different panels.
